# A Nonionic Alcohol Soluble Polymer Cathode Interlayer Enables Efficient Organic and Perovskite Solar Cells

**DOI:** 10.1021/acs.chemmater.1c01430

**Published:** 2021-07-20

**Authors:** Anirudh Sharma, Saumya Singh, Xin Song, Diego Rosas Villalva, Joel Troughton, Daniel Corzo, Levent Toppare, Gorkem Gunbas, Bob C. Schroeder, Derya Baran

**Affiliations:** † King Abdullah University of Science and Technology (KAUST), Physical Sciences and Engineering Division (PSE), KAUST Solar Center (KSC), 23955, Thuwal, Saudi Arabia; ‡ Department of Chemistry, University College London, London WC1H 0AJ, United Kingdom; § Middle East Technical University (METU), Department of Chemistry, 06800 Ankara, Turkey; ⊥ ODTU GUNAM, Middle East Technical University, 06800 Ankara, Turkey

## Abstract

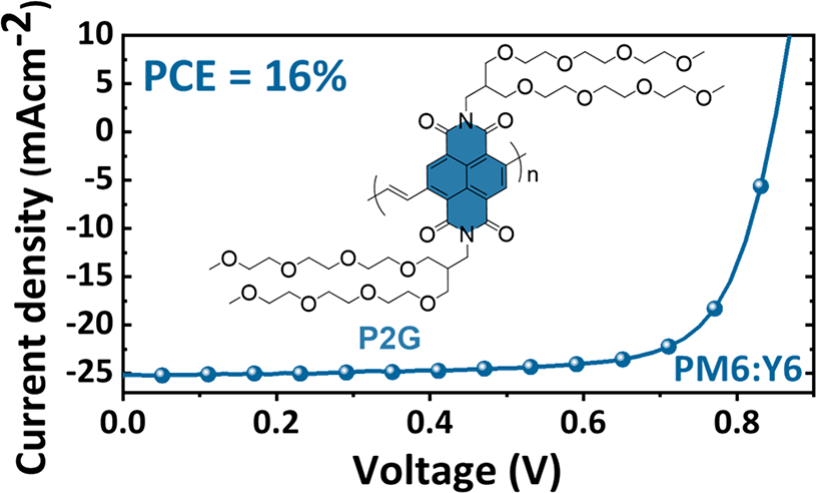

The choice of interfacial materials and their properties play a critical role in determining solar cell performance and stability. For compatibility with roll-to-roll printing, it is desirable to develop stable cathode interface layers (CILs) that can be processed over the photoactive layer using orthogonal solvents. In this study, an *n*-type naphthalene diimide core and oligo (ethylene glycol) side-chain-based conjugated polymer is reported as a universal, efficient CIL for organic and perovskite photovoltaics. Besides good thermal stability and easy processing in alcohol/water, the new CIL is found to possess electron transport properties with an electrical conductivity of 2.3 × 10^–6^ S cm^–1^, enabling its use as a CIL with a film thickness of up to ∼35(±2) nm. Utilizing the new CIL, 16% power conversion efficiency (PCE) is achieved for organic solar cells (OSCs) based on the PM6-Y6 photoactive layer (8.9% PCE for no CIL and 15.1% with state-of-the-art CIL, PDINO), and perovskite solar cells from methylammonium lead iodide yielded a PCE of 17.6%. Compared to the reference devices, the new CIL reduced trap-assisted carrier recombination and increased the built-in potential by 80 mV, simultaneously enhancing all photovoltaic parameters. Moreover, new CIL based devices had better photostability with no burn-in losses.

## Introduction

The field of organic solar cells (OSCs) has witnessed tremendous progress over the past two decades owing to their immense potential to be lightweight, solution-processable, mechanically flexible and their ability to be semitransparent.^1^ The emergence of nonfullerene acceptor (NFA) materials has led to significant improvement in OSCs’ performance,^2−5^ with power conversion efficiency (PCE) crossing the 18% mark for single-junction OSCs.^6^

Besides the development of novel active layer materials, interface engineering has played a significant role in enhancing OSCs’ performance and stability.^7^ Charge transport layers are commonly used to form an ohmic contact between the electrodes and the photoactive bulk heterojunction (BHJ) layer to enable unipolar extraction of charges.^8^ PEDOT:PSS, owing to its ease of processing^9^ and good electrical properties, has been one of the most commonly used hole transporting materials at the anode interface even though its intrinsically acidic and hygroscopic nature remains an issue for device stability.^10,11^ On the other hand, besides the low work function transition metal oxides,^12^ lately, organic materials^13^ have also been utilized as cathode interface layers (CILs). However, the developmental efforts in this direction are still underway, with a focus on designing solution-processable CIL materials that can be deposited over the BHJ layer using orthogonal solvents such as water and alcohols.^14^

**Figure 1:**
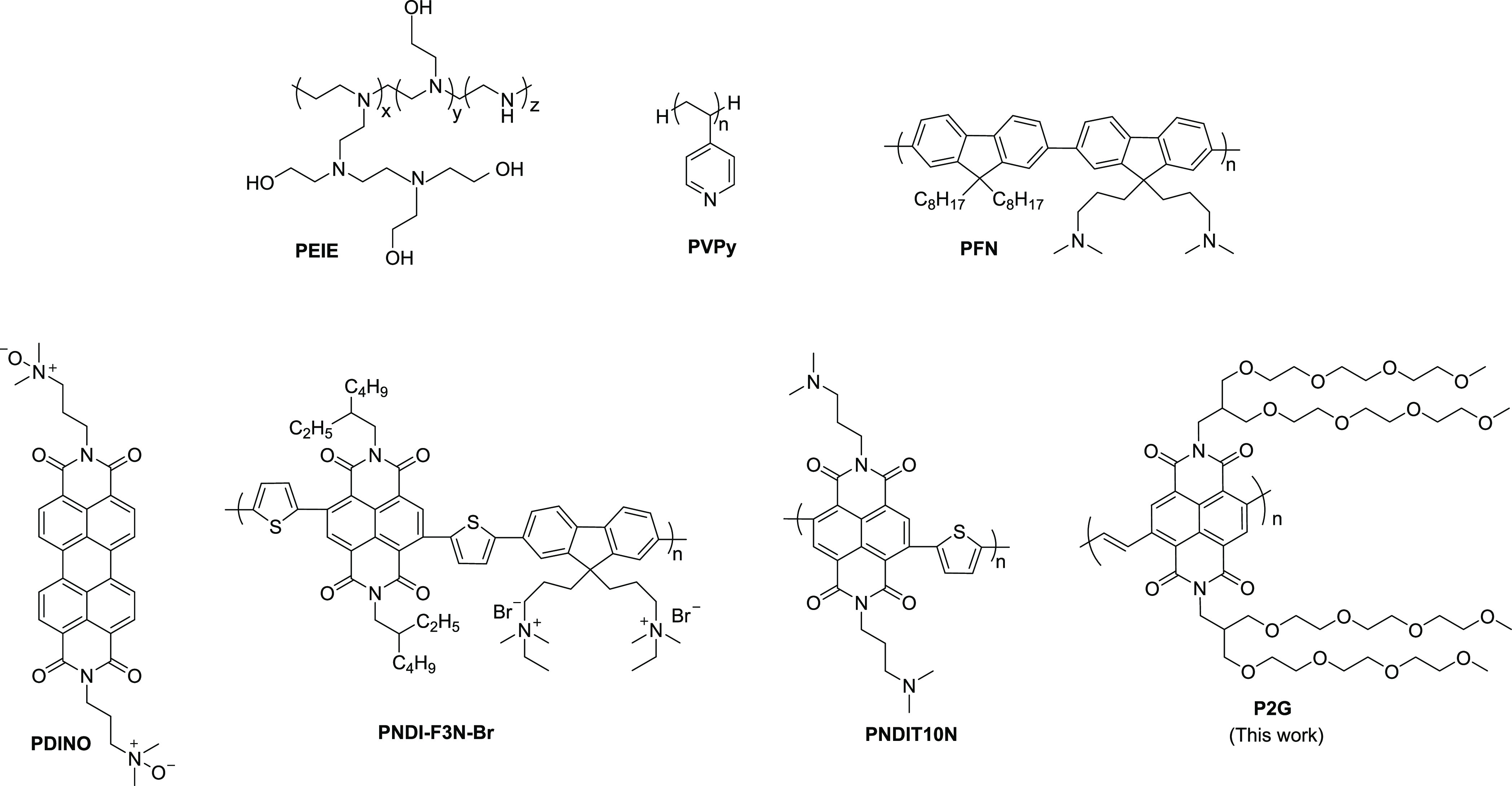
Chemical structures of various materials used as CILs in OSCs.

In the past few years, a range of alcohol processable organic materials including nonconjugated polymers (such as polyethylenimine ethoxylated (PEIE), polyvinylpyridine (PVPy) and polyethylene glycol (PEG),^15−17^ conjugated polymers (for example PNDI-F3N–Br and PNDIT10N),^18,19^ and small molecules (such as Phen-NaDPO and PDINO;^20,21^Figure 1) have emerged as CILs in OSCs. Polyfluorenes such as poly[(9,9-bis(3″-(N,N-dimethylamino)propyl)-2,7-fluorene)- alt-2,7-(9,9-dioctylfluorene)] (PFN) that have side chains with tertiary amine functionality have shown improvement in PCE when employed as a CIL.^22^ In addition to neutral side chains, polymers containing side chains with ionizable functionality^14^ and zwitterionic side chains^23^ have also been used as CILs. Ultrathin interface layer of polyfluorene (PFN) or aliphatic polymers such as polyethylenimine (PEI) with a thickness of 5 nm or less reduce the work function of cathode electrodes by forming interfacial dipoles at the cathode and improve device performance by enhancing built-in potential across the device.^16^ However, the thickness of such CILs based on polyfluorenes and aliphatic polymers such as PEIE are often limited to less than 5–10 nm^24^ due to the intrinsically p-type or insulating nature of these polymers, respectively. N-type small molecule-based interlayers containing perylene diimide (PDI) derivatives have been shown to work efficiently;^20,25^ however, their processability and stability are problematic as they tend to aggregate in the solution,^25^ thus leading to inhomogeneous films both in composition and continuity.

To fabricate solar cells by roll-to-roll printing techniques, it is desirable to employ a conjugated polymer-based CIL that can facilitate charge transport over a wide range of film thicknesses. Moreover, the CIL material should have orthogonal solubility compared to the active layer to enable layer-by-layer sequential processing. In the case of CILs based on polyelectrolytes, though the charged side chains facilitate the dissolution in polar solvents, the counterions can be detrimental to the device’s stability due to the migration of counterions from the interface to the active layer.^26^ The other essential requisite for an ideal CIL is its ability to effectively reduce the electrode work function and enable energetically selective transport of electrons from the active layer to the cathode and to block any hole transport.

In this work, we have developed an alcohol/water-soluble *n*-type polymeric CIL, which does not require charged side chains with mobile counterions for solubility. The new polymer is based on a naphthalene diimide (NDI) core and branched oligo (ethylene glycol) side chains, facilitating electron transfer over film thicknesses of up to 35(±2) nm. OSCs utilizing the new polymeric CIL demonstrate suppressed trap-assisted carrier recombination and enhanced built-in potential, achieving a PCE of 16% for PM6:Y6 based OSCs as compared to 15.1% for the state-of-the-art CIL PDINO. OSCs incorporating the new CIL had better photostability compared to PDINO with no burn-in loss. The new polymer CIL’s applicability is also demonstrated for hybrid perovskite solar cells, where a 17% increase in PCE of MAPI based devices was observed reaching a PCE of 17.6%.

## Results and Discussion

### Synthesis and Characterization

**Scheme 1:**
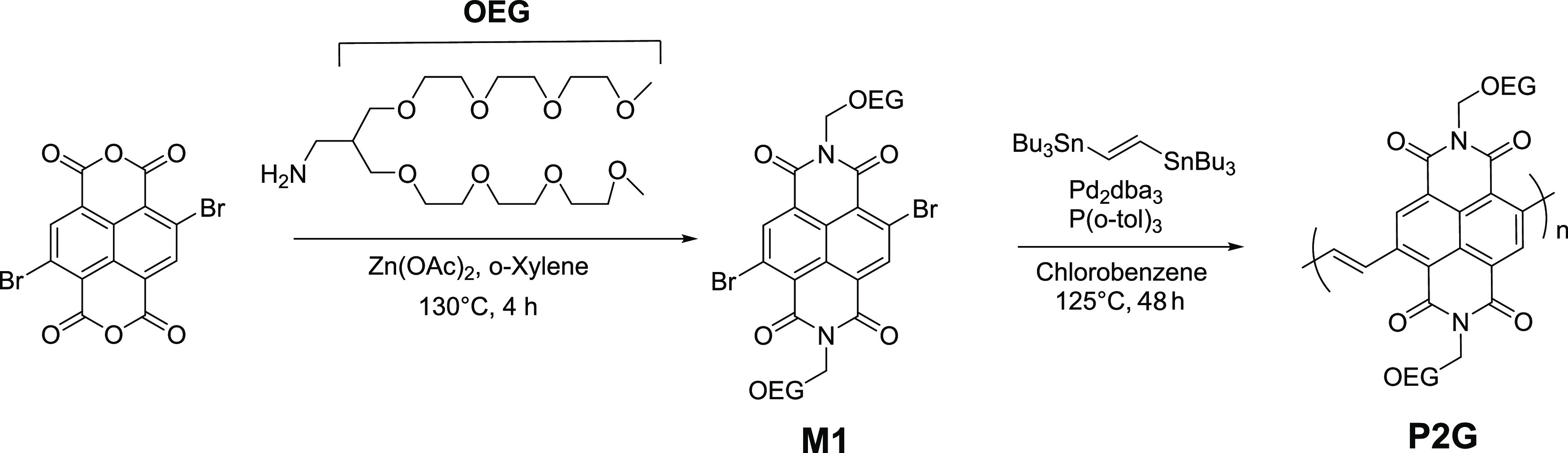
Synthesis of Oligo(ethylene glycol) (OEG) Side-Chain Substituted Monomer M1 and Polymer P2G

The branched oligo(ethylene glycol) (OEG) side-chain substituted NDI monomer was synthesized to obtain a desirable solubility of the polymer in the polar target solvents. The OEG substituted NDI monomer has previously been used for the synthesis of water/alcohol soluble *n*-type conjugated polymers based on the NDI-thiophene backbone.^27^ The synthesis of monomer *N*,*N*′-bis(Teg_2_)-2,6-dibromonaphthalene-1,4,5,8-bis(dicarboximide) (M1) is presented in Scheme S2 (Supporting Information). The synthesis of the branched OEG side chain 1 was carried out according to the literature procedure (Scheme S1).^28,29^ The N-functionalization of the dibromo NDA core was performed in *o*-xylene in the presence of zinc acetate.^30^ M1 was found to be soluble in a range of solvents such as chloroform, dichloromethane, ethyl acetate, methanol, and ethanol. In addition to organic solvents, M1 showed very good solubility in water, which makes it an excellent monomer for the synthesis of potentially water-soluble polymers. M1 was copolymerized with *trans*-1,2-bis(tributylstannyl)ethene via Stille coupling polymerization (Scheme 1).

Since M1 is soluble in polar solvents, solvent selection for polymer purification was crucial. Unreacted monomers and oligomers were removed by hexane and solvent mixtures of hexane/acetone (1:1) and hexane/ethanol (4:1). After the purification process, polymer P2G was obtained (*M*_n_ = 6.8 kg·mol^–1^, *M*_w_ = 12.5 kg·mol^–1^) in 45% yield. The modest molecular weights of this polymer were due to difficulties in purifying the stannyl monomer, leading to minor stoichiometric imbalances between the monomers, thus limiting the molecular weight during the step-growth polymerization.

Branched oligo(ethylene glycol) substituted polymer P2G showed good solubility in chlorinated solvents as well as in alcohols (e.g., methanol, ethanol). The first essential requirement for a polymeric cathode material is to provide orthogonal solubility to other material layers in the organic solar cell fabrication process. P2G can be readily dissolved in ethanol at room temperature with a concentration of 10 mg mL^–1^. Moreover, P2G is also soluble in water/alcohol mixtures without the introduction of any additives. Due to this excellent solubility, homogeneous films of desirable thickness can be coated on top of the active layer in OSC devices.

**Figure 2:**
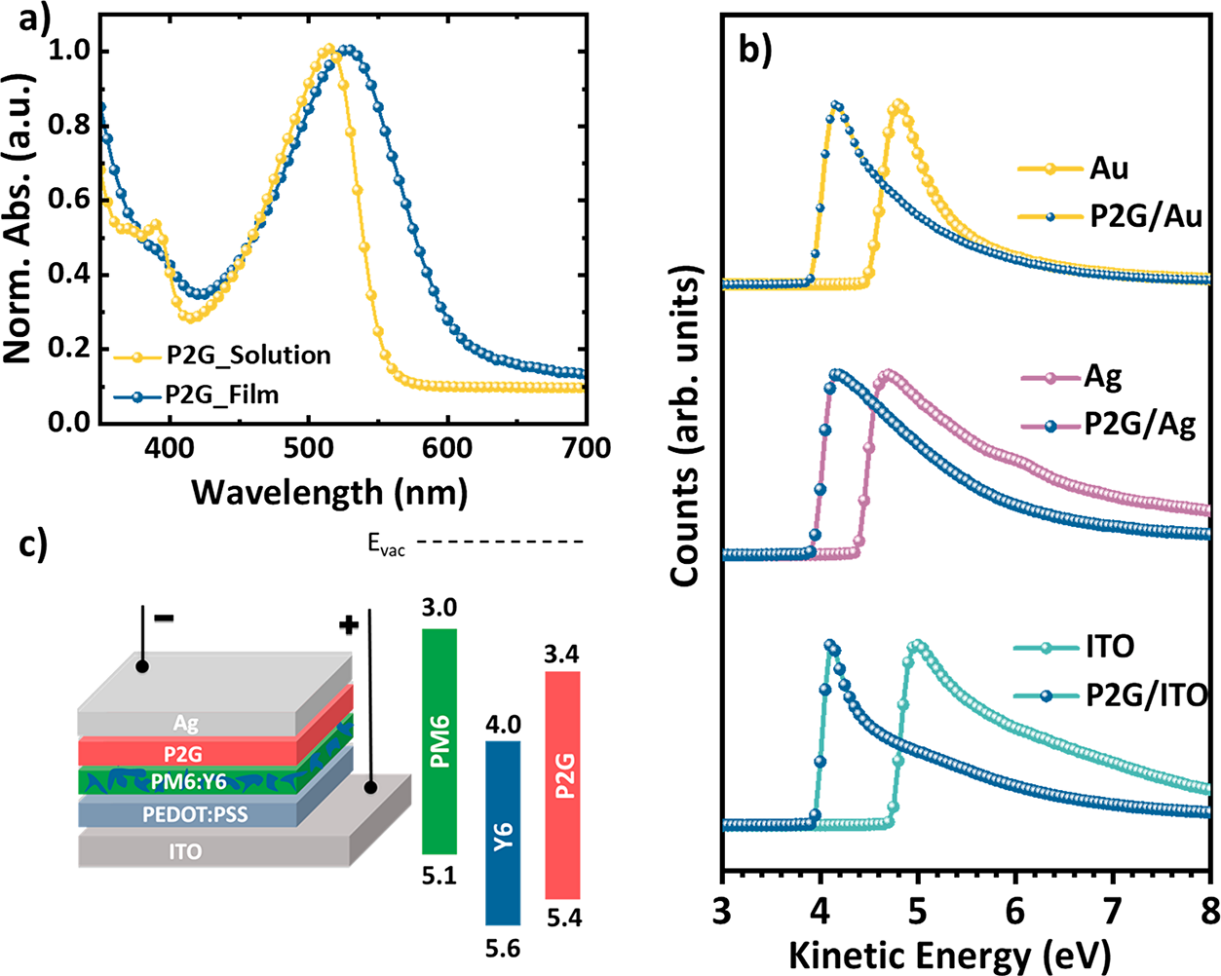
(a) Normalized UV–vis absorption spectra of P2G in solution and thin film; (b) secondary electron cutoff of the UP spectra depicting the change in the work function of P2G modified ITO, Ag, and Au electrodes; (c) schematic showing the device structure and the energy levels of the state-of-the-art donor (PM6), acceptor (Y6), and P2G, measured using UPS and LE-IPES (refer to SI for details).

UV–vis absorption spectra of P2G in chloroform solution were recorded (Figure 2a), and an absorption maximum (λ_max_) of 515 nm was observed. A chloroform solution of P2G at a 10 mg mL^–1^ concentration was spin-coated onto a glass substrate, and the absorption spectrum of the thin film was recorded. The λ_max_ for P2G film coated from chloroform showed a 13 nm red shift compared to the solution spectrum, indicative of aggregation of the polymer chains in the solid state. The optical bandgap was calculated from thin-film absorption band onset as 2.02 eV. The choice of the vinylene spacer with the NDI core in P2G resulted in a relatively narrow absorption range, which would be advantageous for its use as a CIL.

Frontier orbital energy levels of P2G were calculated by combining cyclic voltammetry experiment (in solution) data and optical bandgap (Figure S7). The cyclic voltammograms (CVs) of P2G were recorded in 0.1 M tertrabutylammonium hexafluorophosphate dissolved in degassed anhydrous dichloromethane. The first reduction onset potential was used to determine an electron affinity (EA) of −4.03 eV for P2G. The ionization potential was calculated at −6.05 eV by subtracting the optical bandgap from the LUMO energy level. The P2G EA is close to the EA typically measured for NFA materials^31^ and is therefore expected to facilitate electron transfer efficiently between the acceptor and the cathode. Unlike PEIE or PFN, the intrinsically *n*-type nature of P2G would be beneficial in not limiting its thickness to <5–10 nm, when used as a CIL.

The thermal stability of P2G was studied by thermogravimetric analysis (Figure S8a). P2G was found to be stable up to 300 °C, with a 5% weight loss observed after 350 °C. To study the melting and crystallization behavior of P2G, a differential scanning calorimetry (DSC) thermogram was recorded between −20 and 350 °C (Figure S8b). OEG substituted polymer P2G did not show any significant thermal transition in this temperature range.

The ability of the CIL to modify the electrode work function is often associated with the interface dipole induced by the CIL.^16,18^ Ultraviolet photoelectron spectroscopy (UPS) measurements were therefore used to probe the work function modification of different electrodes of ITO, Ag, and Au. A thin layer (5 nm) of P2G was found to effectively reduce the work function of ITO from 4.73 to 3.94 eV (Figure 2b), and the work function was found to be largely unchanged for P2G thicknesses of up to ∼35(±2) nm (Figure S9a). P2G modified Ag and Au electrodes were also found to have a low work function of 3.92 and 3.91 eV, respectively. This demonstrates that P2G can induce a large dipole on a variety of electrode surfaces, significantly reducing the work function. Unlike P2G, polymer P2 (Scheme S3) with the same backbone as P2G but functionalized with alkyl side chains instead of the OEG side chains was found to only marginally change the electrode work function by ∼0.1 eV (Figure S9b). Thus, the strong dipole induced by P2G at the electrode interface is attributed to the polar OEG side chains. The induced dipole between the P2G and electrode interface would provide an energetically favorable electron transport from the active layer to the electrode and increase the built-in potential across the device.^32^

Direct measurement of ionization energy (IE) and electron affinity (EA) of thin films of organic semiconductors is more representative of their corresponding energetics in OSC devices. Therefore, UPS and low-energy inverse photoelectron spectroscopy (LEIPES) were used to measure the IE and EA of P2G (Figure S10c) and were found to be 5.41 and 3.38 eV (Figure 2c), respectively. The difference between the frontier molecular orbital energies measured using photoelectron spectroscopy and electrochemistry could be due to the differences in molecular packing and ordering in solid thin films compared to that in solution. Attempts have been made to better understand and explain such discrepancies,^33^ and their implications are currently a topic of discussion in the OSC community.^31^

### 2.2 Photovoltaic Performance in OSCs

To evaluate the photovoltaic performance of P2G CIL in OSCs, photovoltaic devices with the structure ITO/PEDOT:PSS ((poly(3,4-ethylenedioxythiophene):poly(styrene-sulfonate)) (20 nm)/ photoactive layer/P2G/Ag (80 nm) were fabricated. First, the photoactive layer based on a PTB7-Th:IEICO4F (chemical structures in Figure S11) BHJ blend (∼130 nm, 1:1.5 in chlorobenzene with 4% v/v of 1-chloronapthalene) was used to check the efficacy of P2G as a CIL. OSCs incorporating a P2G film with an optimum thickness of 5(±2) nm (measured using atomic force microscopy) were found to have a PCE of 10.5%, with a high short-circuit current density (*J*_sc_) of 23.6 mA cm^–2^ and an open-circuit voltage (*V*_oc_) of 0.71 V (Table 1), comparable to that previously reported for PTB7-Th:IEICO-4F devices.^34,35^ The effect of P2G film thickness on OSCs’ performance was examined by varying the thickness from <5 nm up to 35 nm. Notably, the performance of P2G-based devices was found to be only slightly sensitive to the thickness (Figure S11b). For example, OSCs with a 35 nm P2G film retained a PCE of 9.3%, unchanged *V*_oc_ of 0.71 V, and a slightly increased series resistance of 2.7 ohm cm^2^ (from 1.8 ohm cm^2^ for 5 nm P2G devices). Though the increasing P2G thickness led to a slight reduction in the *J*_sc_, devices with 35 nm of P2G still resulted in a *J*_sc_ of 20.9 mA cm^–2^, closely matching the integrated *J*_sc_ from the EQE measurements (Figure S11c).

**Table 1:** Photovoltaic Parameters of Conventional Devices Based on PTB7-Th:IEICO-4F and PM6:Y6 Active Layer under AM 1.5 G Illumination, 100 mW cm^–2^^a^

BHJ	CIL	thickness [nm]	*J*_sc_ [mA cm^–2^]	integrated *J*_sc_ [mA cm^–2^]	*V*_oc_ [V]	FF [%]	PCE [%]	av. PCE [%]
PTB7-Th: IEICO-4F	P2G	∼1–3	22.9	22.9	0.70	60.2	9.8	9.7
		5	23.6	23.0	0.71	62.2	10.5	10.5
		15	22.0	21.9	0.71	62.4	9.9	9.6
		35	20.9	21.0	0.71	62.7	9.3	9.3
PM6:Y6	P2G	5	25.2	24.4	0.85	74.8	16.0	15.7
	PDINO	8	24.7	24.0	0.84	72.9	15.1	14.8

a Integrated *J*_sc_ was within a 5% deviation of the *J*_sc_ value acquired from *J*–*V* curves. Average values calculated over 10 devices.

Further, the applicability of P2G as a CIL in scalable fabrication methods, e.g., inkjet printing,^36^ was investigated to print the layers. Devices based on ITO-glass incorporating a spin-coated PEDOT:PSS HTL and PTB7-Th:IEICO-4F active layer and a thin P2G CIL inkjet-printed (see SI for experimental details) in ambient air atmosphere were fabricated. A PCE of 9.2% was achieved (Figure S12), with a *V*_oc_ of 0.70 V and FF of 58.8%, demonstrating good compatibility of P2G with printing methods of fabrication.

**Figure 3:**
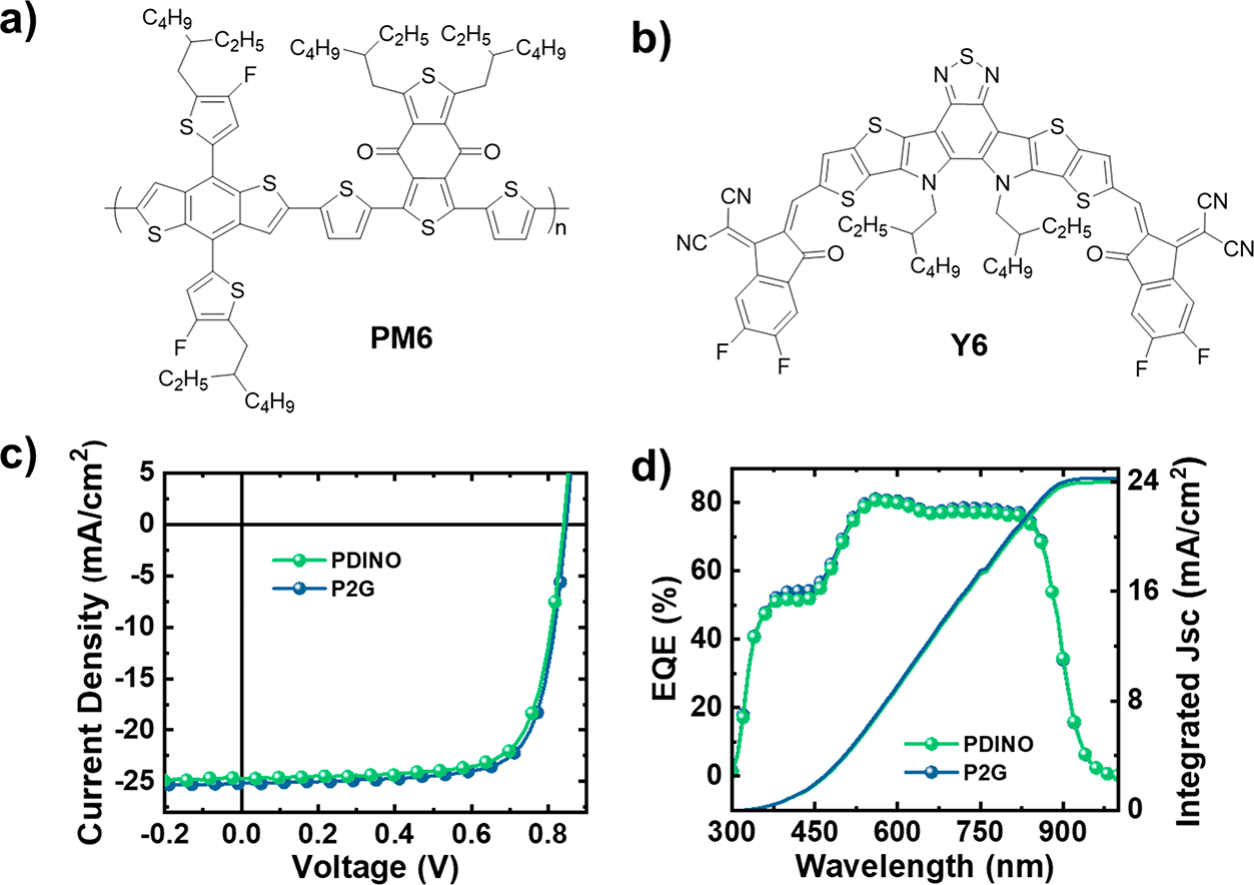
Chemical structure of (a) PM6 and (b) Y6, (c) J-V, and (d) EQE curves of the best-performing PM6-Y6 devices using a PDINO and P2G CIL.

P2G as the CIL was then systematically studied in OSCs with a PM6:Y6 (chemical structure in Figure 3a and b) based photoactive layer (∼140 nm, 1:1.2 in CHCl_3_ with 0.5% v/v 1-chloronaphthalene) and compared with no CIL devices and the state-of-the-art system with the PDINO (chemical structure shown in Figure 1; UPS, LEIPES, and UV–vis shown in Figure S10d and e) as the CIL, which was shown to deliver a high PCE of 15.7%.^4,20^ The device without a CIL only delivered a moderate PCE of 8.91% with a *J*_sc_ of 22.8 mA cm^–2^, *V*_oc_ of 0.66 V, and fill factor (FF) of 58.2% (Figure S13a). Intuitively, the insertion of PDINO as a CIL (Figure 3c, Table 1) significantly improved the PCE to 15.1% (*J*_sc_ = 24.7 mA cm^–2^, *V*_oc_ = 0.84 V, FF = 72.9%), which is similar to that in previously reported work.^4^ However, when PDINO was replaced with P2G as the CIL, the champion PCE of 16.0% was achieved, accompanied by a simultaneous enhancement in all device parameters (*J*_sc_ = 25.2 mA cm^–2^, *V*_oc_ = 0.85 V, FF = 74.8%).

To gain further insight into the impact of P2G on the light-to-current conversion, external quantum efficiency (EQE) measurements were performed. Figure 3d shows the EQE spectra of devices incorporating P2G and PDINO as CILs. In both cases, the maximum EQE plateau reached around 75–80% between 500 and 850 nm. The *J*_sc_ values were within 5% of the integrated *J*_sc_ values calculated from the EQE spectral curves (Table 1), confirming the observed enhancement of *J*_sc_ when P2G is used as the CIL.

**Figure 4:**
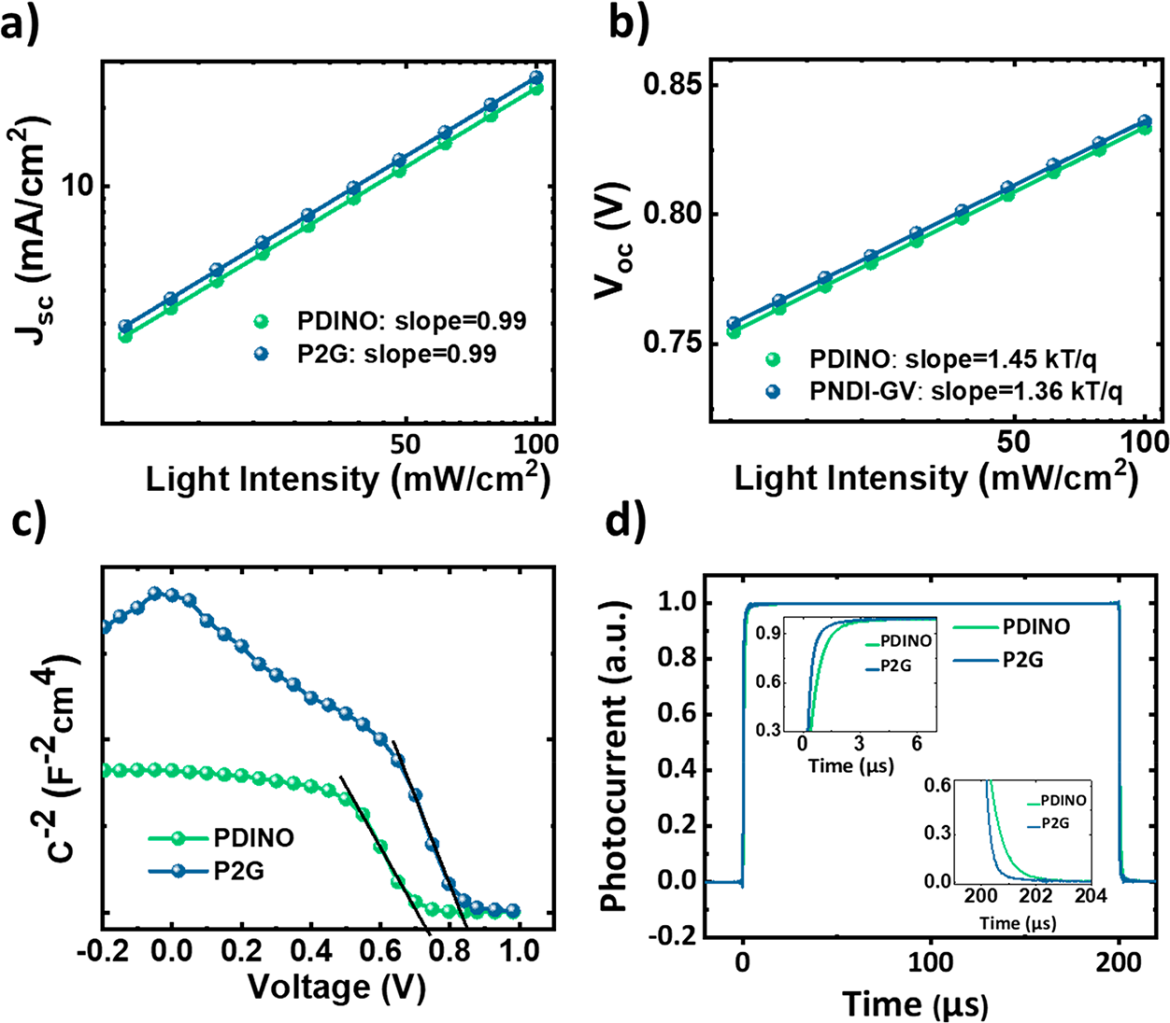
Light intensity-dependent (a) *J*_sc_ and (b) *V*_oc_ measurements. (c) Mott–Schottky characteristics [C^2–^ (V)] and (d) normalized TPC plots of PM6:Y6 based solar cells with PDINO and P2G CILs. Inset shows the zoomed in view of the change in the rise and fall time of the photocurrent.

To investigate the origin of increased *J*_sc_ and FF for devices incorporating P2G as the CIL, the charge recombination dynamics were probed by measuring *J–V* characteristics at different light intensities. The plots of *J*_sc_ and *V*_oc_ vs the natural logarithm of the light intensity provide information about bimolecular recombination and trap-assisted recombination, respectively.^37^ A slope of less than 1 indicates bimolecular recombination as the primary loss where a slope of more than *kT*/*q* is often associated with trap-assisted recombination. The calculated slopes of the *J*_sc_ vs light intensity for both the PDINO and P2G curves were found to be close to 1 (Figure 4a), implying that the bimolecular recombination is not the main recombination channel when either of the CILs is used. Furthermore, light intensity-dependent *V*_oc_ measurements (Figure 4b) show that the devices with P2G exhibit reduced trap-assisted recombination, as the slope calculated for PDINO and P2G was 1.46 and 1.38 *kTq*^–1^, respectively. This could also account for the relatively higher FF values observed for devices incorporating P2G as the CIL.

To further unravel the enhancement of solar cell performance upon using the P2G as the CIL, the electrical conductivities of PDINO and P2G were measured (Figure S14a) using a two-point probe method (see the Experimental Section for more details). The measured conductivity of PDINO is 9.3 × 10^–6^ S cm^–1^, which is close to that reported by Zhang et al.^20^ Electrical conductivity of the P2G thin film was found to be 2.3 × 10^–6^ S cm^–1^, featuring a comparable electron-transport property to that of PDINO and explains the relatively low sensitivity of device performance to the P2G film thickness as discussed earlier. It is worth noting that these two CIL materials’ electrical conductivity, when processed using methanol, is close to 3 orders of magnitude higher than that of the well-known cathode interlayer PFN (∼6 × 10^–9^ S cm^–1^).^20^ To further understand the origin of high conductivity of P2G, the conductivity of P2G was compared with that of the alkyl side chain polymer P2. When processed using the same solvent, P2G was found to have an order of magnitude higher conductivity compared to P2 (Figure S14b).

The electrical characterization of devices under dark conditions was also performed to further understand the higher *J*_sc_ and FF in P2G-based devices. P2G based devices were found to have improved diode properties (Figure S15), exhibiting lower leakage currents and higher rectification, which could be attributed to the electron selective contact of P2G with the active layer and potentially a more conformal coverage compared to a small molecule film.

DC biased AC impedance measurements were also performed to gain further insight into the improvements in device parameters.^38,39^Figure 4c shows the capacitance (*C*)–voltage (*V*) characteristics attained by applying a low AC perturbation signal with a fixed frequency and sweeping the DC bias. In both cases, a linear relationship is observed at the low forward voltages, with the extrapolated intercept with the voltage axis being related to the flat-band potential. In devices incorporating the P2G CIL, the built-in potential (*V*_bi_) was found to increase from 0.72 to 0.80 V, an increase of 80 mV compared to that in devices utilizing the PDINO. The higher *V*_oc_ observed in the P2G-based solar cells is thus attributed to the increased *V*_bi_ in the device.

Transient photocurrent (TPC) measurements characterized by an initial voltage-dependent sweep-out of mobile carriers, followed by a long, bias-insensitive photocurrent tail, were performed to understand the impact of increased *V*_bi_ on the extraction and recombination of charge carriers. TPC measurements have been previously used to study charge transport and recombination dynamics in fullerene and nonfullerene solar cells.^40−43^ For both PNDIO and P2G, the turn-on and turn-off dynamics were fast (Figure 4d). However, on closer examination (inset of Figure 4d), the rise/fall time (the time taken to go from 10% to 90% response) for devices with the P2G was relatively shorter (0.7 μs) than their PDINO counterparts (1.3 μs). In line with the higher *J*_sc_ values obtained for P2G devices, faster saturation and decay kinetics in the P2G devices (Figure 4d inset) indicate more rapid charge generation and extraction than the PDINO-based ones, and reflect the reduced trap-assisted recombination and enhanced *V*_bi_ observed for these devices.

The surface topography of thin films of PDINO and P2G deposited on the glass as well as on the corresponding PM6:Y6 photoactive layer was investigated (Figure S16) using atomic force microscopy (AFM). The root-mean-square (RMS) roughness of P2G and PDINO was determined to be 2.8 and 7.2 nm, respectively. Consequently, upon deposition on top of the photoactive layer, P2G film was found to have an RMS roughness of 4.3 nm, compared to 8.3 nm for that of the PDINO layer (Figure S16c and d). The relatively rough surface of PDINO compared to that of P2G could be due to strong molecular aggregation known to occur in PDI,^44^ demonstrating better film-forming properties of P2G, which can also explain the reduced leakage currents (Figure S15) observed in these devices.

Last, the photostability of devices incorporating P2G and PDINO CIL was studied. The two devices showed considerable differences in the degree of performance drop, up to a studied period of 1000 h (Figure S13c). Most importantly, devices with PDINO CIL were found to have significant burn-in losses, losing over 30% of the initial PCE in the first 50 h. On the contrary, the P2G-based devices did not demonstrate burn-in losses, and the PCE linearly dropped only 10% of the initial value after >200 h of operation.

To demonstrate the wider applicability of P2G as a CIL, P2G was also utilized in another OSC BHJ system (PM6:ITIC-4Cl) and in hybrid perovskite solar cells. OSCs with a PM6:ITIC-4Cl BHJ blend incorporating a P2G CIL exhibited a PCE of 13.3% (Figure S17, Table S1), comparable to the previously reported PCE for PM6:ITIC-4Cl devices.^45^ This further demonstrates that P2G is a versatile CIL which is compatible with a range of different NFA materials, which are sensitive to chemical interactions with the CILs.^46,47^

**Figure 5:**
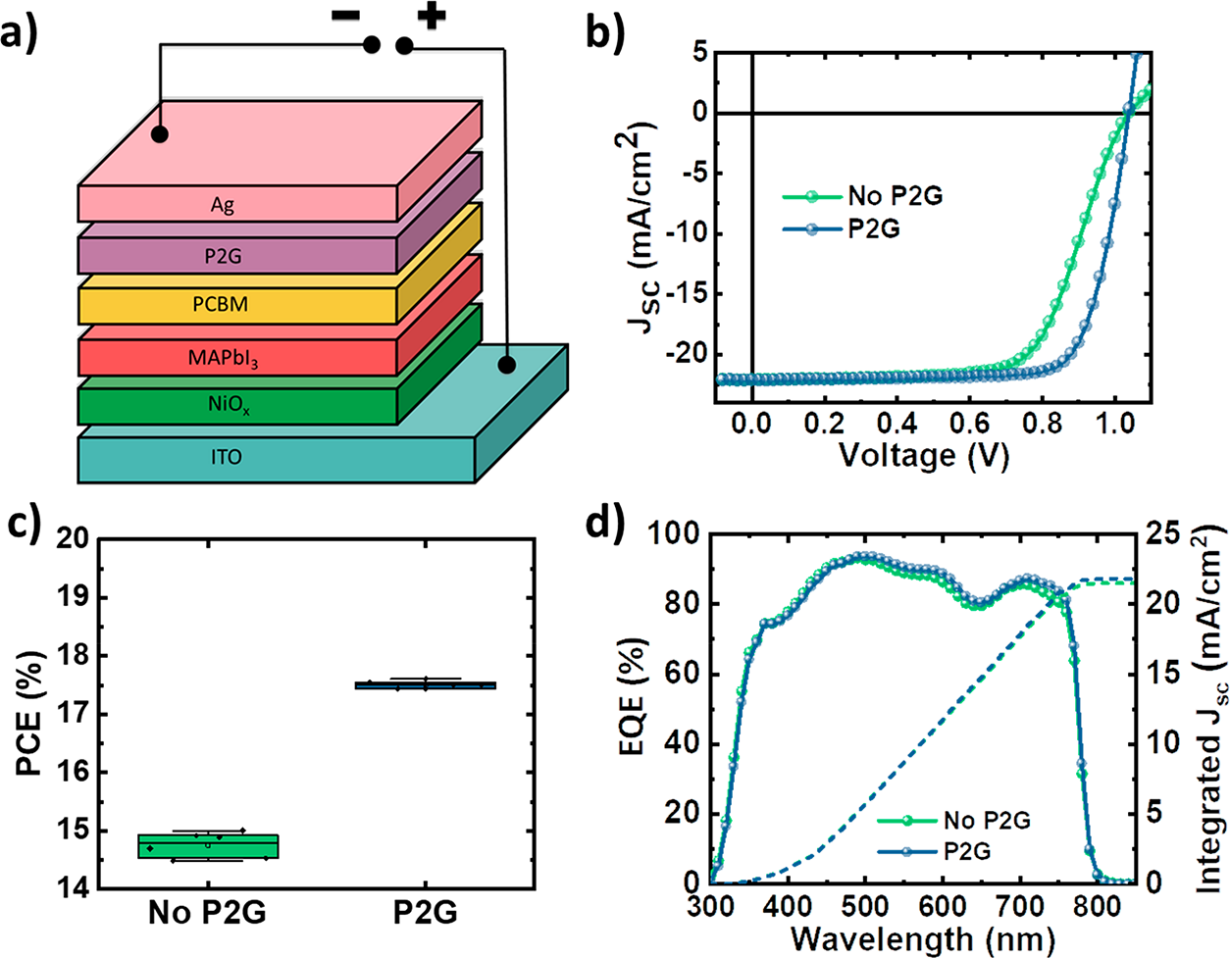
(a) Device structure and (b) *J*–*V* curves of hybrid perovskite solar cells fabricated with and without P2G, (c) box plots showing the PCE along with the standard deviation across six devices with and without P2G, (d) EQE curves of best-performing devices with and without the P2G CIL.

Perovskite solar cells with an inverted p-i-n structure using a NiO_*x*_ hole transport layer (HTL), MAPbI_3_ absorber, and the PC_61_BM electron transport layer (ETL; Figure 5a) were fabricated. A thin layer of small molecules such as bathocuproine (BCP)^8^ or 3-[6-(diphenylphosphinyl)-2-naphthalenyl]-1,10-phenanthroline (Phen-NaDPO)^48^ is commonly used as a CIL in between the ETL and the cathode to improve the ohmic contact. The *J*–*V* curve of control devices without a CIL exhibited an S shape (Figure 5b) potentially originating due to poor electron transport from PC_61_BM to the silver electrode. In the absence of any CIL between fullerene and the metal electrode, a Schottky barrier is known to form, inhibiting the extraction of electrons from the semiconductor to the external circuit.^8,49^ Control devices with only PC_61_BM were found to have a relatively low FF of 65%, resulting from this extraction barrier, leading to a PCE of 15.0%. The introduction of P2G between the PC_61_BM and the silver electrode was found to nullify this barrier, eliminating the s shape and significantly increasing the FF of the device from 65% to 77%. This resulted in a maximum PCE of 17.6%, surpassing a PCE of 15% for the PC_61_BM reference device (Figure 5c). The EQE spectra of both of the devices were found to be similar (Figure 5d), and the integrated current density was in agreement with the measured *J*_sc_ of devices with and without P2G (Table S2). It is worth noting that perovskite solar cells’ performance utilizing P2G as the CIL is also significantly better than those of devices using a comparable solution-processed Phen-NaDPO CIL, reported earlier.^48^

On the basis of the above findings, it is evident that P2G can be successfully applied as a versatile CIL to OSCs as well as hybrid perovskite solar cells and consistently deliver significant improvements to the device performance.

## Conclusions

In summary, a universal NDI-based *n*-type conjugated polymer with a vinylene linker and oligo (ethylene glycol) side chain was synthesized as a CIL and used in both organic and hybrid perovskite solar cells. P2G is alcohol soluble, has good thermal stability of up to 300 °C, has appropriate frontal molecular energy levels, and possesses a high electrical conductivity of 2.3 × 10^–6^ S cm^–1^.

P2G was demonstrated as a versatile CIL material that can effectively modify the work function of a range of electrode materials, can be deposited via printing methods, and can be used successfully with photoactive layers based on several high-performing donor–acceptor blends. Owing to its high electrical conductivity, a P2G CIL of up to 35 nm was demonstrated to work efficiently. The device performance showed little sensitivity to the P2G film thickness, making it particularly promising for applications in roll-to-roll fabrication of OSCs.

OSCs based on the PM6-Y6 photoactive layer and utilizing a P2G CIL resulted in a considerably improved PCE of 16% compared to 15.1% for devices having PDINO as the reference CIL. This improvement was attributed to the reduction of trap-assisted recombination and increased built-in potential by 80 mV, facilitating faster charge extraction. Moreover, OSCs based on P2G were burn-in free, with enhanced photostability as compared to PDINO. P2G was also demonstrated to be an efficient CIL for the reduction of interfacial energy barriers in hybrid perovskite solar cells, resulting in a PCE of 17.6% for MAPbI_3_-based p-i-n planar devices compared to a PCE of 15% in reference devices.

The efficacy of P2G CIL indicates that oligo functionalization of the NDI-based polymers is a promising strategy to achieve alcohol/water-soluble polymer interlayers with desired electrical and electronic properties.

## Experimental Section

### Synthesis

All reagents and anhydrous solvents were purchased from commercial sources and used as received unless otherwise noted. ^1^H NMR and ^13^C NMR were recorded in CDCl_3_ in 400 MHz and 700 MHz NMR spectrometers. High-resolution mass spectrometric measurements were carried out using the ESI method.

#### Polymer P2G

In a 20 mL microwave vial, monomers N,N′-bis(Teg_2_)-2,6-dibromonaphthalene-1,4,5,8-bis(dicarboximide) (M1, 400 mg, 0.338 mmol) and *trans*-1,2-bis(tributylstannyl)ethene (205 mg, 0.338 mmol) were dissolved in 15 mL of anhydrous, degassed chlorobenzene. Pd_2_(dba)_3_ (2 mol %) and P(*o*-tol)_3_ (8 mol %) were added to the solution, and the reaction mixture was purged with nitrogen for 10 min. The vial was sealed and heated to 125 °C for 48 h. For end-capping, a chlorobenzene solution of 2-(tributylstannyl)thiophene (5 mol %) and Pd_2_(dba)_3_ were added to the vial, and the reaction mixture was heated for 2 h followed by the addition of 2-bromothiophene (10 mol %). The reaction was continued for another 2 h. After end-capping, the reaction mixture was cooled to room temperature. The crude reaction mixture was then precipitated in cold hexane. The solid obtained was directly filtered into a glass-fiber thimble and washed with a cold hexane/acetone (1:1) solvent mixture to remove the unreacted glycol substituted NDI monomer. Soxhlet extraction was carried out with hexane for 12 h to remove unreacted *trans*-1,2-bis(tributylstannyl)ethene monomer, followed by a solvent mixture of hexane/ethanol (4:1) to remove unreacted monomers and oligomers. Final extraction was done with chloroform to collect the polymer. The chloroform fraction was then passed through a short Celite plug to remove the catalyst. The chloroform fraction was further concentrated under reduced pressure and precipitated from cold hexane.

P2G (160 mg, 45%) ^1^H NMR (400 MHz, CDCl_3_) δ: 9.22 (broad s, 2H), 9.00 (broad s, 2H), 4.37 (broad s, 4H), 3.59- 3.50 (m, 56H), 3.34 (s, 12H), 2.63 (broad s, 2H).

GPC (dichlorobenzene, 80 °C): *M*_n_ = 6,863 g·mol^–1^, *M*_w_ = 12,490 g·mol^–1^, and *Đ* = 1.82.

### UV–vis

Absorption spectra were recorded at room temperature in a quartz cuvette and on glass substrates using a Shimadzu UV3600 UV–vis–NIR spectrometer.

### TGA

Thermogravimetric analysis (TGA) was performed under nitrogen on a TA Instruments Q500 at a heating rate of 10 °C/min.

### DSC

Differential scanning calorimetry (DSC) was conducted on a PerkinElmer DSC4000 DSC instrument under nitrogen at a heating/cooling rate of 10 °C/min.

### CV

Cyclic voltammograms were recorded using an Autolab PGSTAT101 potentiostat with a standard three-electrode system. A glassy carbon electrode was used as a working electrode, Pt wire as a counter electrode, a Ag/Ag^+^ electrode as the standard electrode, and Fc/Fc^+^ as an internal standard. The measurements were carried at a scan rate of 100 mV/s.

### AFM

The surface and topography of the active layer have been measured by atomic force microscopy (NT-MDT Solver Next)

### Ultraviolet Photoelectron Spectroscopy (UPS)

UPS measurements were performed in an ultrahigh vacuum chamber (base pressure of 10^–10^mbar) equipped with a Sphera II EAC 125 7-channeltron electron analyzer calibrated with the Fermi edge of clean polycrystalline silver. The spectra were recorded using the He I line (excitation energy of 21.22 eV) at pass energy of 10 eV, with −10 eV of an external bias. The work function of the samples was determined from the secondary electron cutoff of the UP spectra, as described elsewhere.^50^

### Low Energy Inverse Photoelectron Spectroscopy (LEIPES)

Measurements were performed in isochromatic mode using an ultrahigh vacuum (base pressure 10^–9^ mbar) setup built in-house, as described elsewhere. The emitted photons were detected using a solid-state PMT detector (Hamamatsu R585) mounted outside of a vacuum and equipped with a band-pass filter of 280 nm (Semrock) with a narrow wavelength window of 10 nm. Samples were measured immediately after the UPS measurements by transferring to the LEIPES manipulator within an ultrahigh vacuum atmosphere without air exposure. The onset energy of the occupied and unoccupied frontal molecular orbitals was estimated by deconvolution of the spectra using Gaussian functions and a Taugaard background.

### Device Fabrication

Patterned ITO substrates (10 Ω sq^–1^, Xinyan Technologies) were sequentially sonicated in detergent, deionized water, acetone, and isopropyl alcohol before being dried under N_2_ and treated with oxygen plasma for 10 min. OSCs were fabricated with the conventional device structure ITO/PEDOT:PSS: HJ/CIL/Ag. A thin layer of PEDOT:PSS (CLEVIOS P VP AI 4083) was spin-coated at 4000 rpm for 30 s on clean ITO substrates. The PEDOT:PSS coated substrates were then annealed at 160 °C for 10 min in the air and subsequently transferred to a nitrogen-filled glovebox for further fabrication. The active layer in the case of PTB7-Th:IEICO-4F (both supplied by one-materials) devices was deposited from a 25 mg mL^–1^ solution of PTB7-TH:IEICO-4F (1:1.5) in chlorobenzene (with 4% v/v of 1-chloronapthalene) spin-coated at 2500 rpm for 2 min. The PM6:Y6 (supplied by 1-material) active layer was deposited from a 16 mg mL^–1^ solution of PM6:Y6 (1:1.2) in CHCl_3_ with 0.5% v/v 1-chloronaphthalene spin-coated at 2500 rpm. For PM6:ITIC-4Cl (1-material) devices, the active layer was spin-coated (1:1 D–A solution, 20 mg mL^–1^ in chlorobenzene with 0.5% v/v 1-chloronaphthalene as the solvent additive) at 2000 rpm for 30 s. CIL was deposited from a methanol solution of either PDINO (1 mg mL^–1^) or P2G (0.5 mg mL^–1^), followed by 100 nm of Ag thermally evaporated using a shadow mask, defining the geometrical active area of 0.1 cm^2^.

Perovskite solar cells were fabricated according to a previous report.^8^ Briefly, a solution containing 0.3 M nickel acetate tetrahydrate and 0.3 M ethanolamine in 2-methoxyethanol was spin-coated onto cleaned ITO substrates, followed by thermal annealing at 250 °C for 30 min to form a NiO hole-selective layer. A perovskite precursor solution was made by dissolving 1.25 M PbI2 (Alfa Aeser) and 1.25 M methylammonium iodide (Greatcell Solar) in a 4:1 vol mixture of N,N-dimethylmethanamide and dimethyl sulfoxide. Perovskite films were spin-coated onto NiO at 4000 rpm for 30 s inside a glovebox, with 150 μL of chlorobenzene deposited onto spinning substrates 15 s from the end of the cycle. Films were then annealed at 100 °C for 20 min. A solution of 20 mg mL^–1^ PC_61_BM in chlorobenzene was spin-coated onto cooled perovskite films at 1300 rpm for 30 s to form an electron-selective layer. In the case of P2G containing samples, a 0.5 mg mL^–1^ solution of P2G in methanol was spin-coated onto PC_61_BM films dynamically at 3000 rpm before drying at 50 °C for 10 min. The 100-nm-thick silver electrodes were deposited via evaporation through a shadow mask at 10^–6^ mBar. Perovskite solar cell *J*–*V* measurements were performed from *J*_SC_ to *V*_OC_ at a rate of 20 mV^–1^s.

### Electrical Characterization

Electrical conductivity was measured by the two-point probe method. Gold lines (30 nm thick, 1000 μm wide, and length (*L*) of 30, 40, 50, 80, and 100 μm) were thermally evaporated on the bare glass to serve as bottom contact electrodes. P2G and PDINO were spin-coated on top of the gold electrodes, following the same procedure as for the PV devices. The conductivity was calculated by measuring the resistance with a Keithley 4200-SCS and a probe station within a nitrogen flow box. The thickness of the films was determined by AFM (Solver Next by NT-MDT).

### Impedance Spectroscopy (IS)

The IS measurements were implemented using a Hewlett-Packard 4284A precision LCR meter. The frequency range was from 20 Hz to 1 MHz, and the amplitude of the oscillating signal was 5 mV. The obtained data were fitted with Scribner Associates Z-View software v2.6 in terms of appropriate equivalent circuits.

### Transient Photocurrent (TPC)

Devices were illuminated inside a nitrogen-filled glovebox with a 405 nm laser diode for 200 μs, sufficient to reach a constant open circuit voltage with steady-state conditions. At the end of the illumination period, an analog switch was triggered that switched the solar cell from open-circuit to short-circuit (50 ω) conditions within less than 50 ns.

## Supporting Information Available

Synthesis scheme of monomer and polymer, NMR spectra, cyclic voltammetry scans, thermogravimetric analysis curves, differential scanning calorimetry scans, UPS and LEIPES spectra, J-V and EQE plots, device stability data, electrical conductivity, AFM images, solar cell performance parameters (PDF)

## Abbreviations

**PTB7-Th**: Poly[4,8-bis(5-(2-ethylhexyl)thiophen-2-yl)benzo[1,2-b;4,5-b′]dithiophene-2,6-diyl-*alt*-(4-(2-ethylhexyl)-3-fluorothieno[3,4-*b*]thiophene-)-2-carboxylate-2–6-diyl)]
**PM6**: Poly[(2,6-(4,8-bis(5-(2-ethylhexyl-3-fluoro)thiophen-2-yl)-benzo[1,2-b:4,5-b′]dithiophene))-*alt*-(5,5-(1′,3′-di-2-thienyl-5′,7′-bis(2-ethylhexyl)benzo[1′,2′-c:4′,5′-c′]dithiophene-4,8-dione)]
**IEICO-4F**: 2,2′-[[4,4,9,9-Tetrakis(4-hexylphenyl)-4,9-dihydro-s-indaceno[1,2-b:5,6-b′]dithiophene-2,7-diyl]bis[[4-[(2-ethylhexyl)oxy]-5,2-thiophenediyl]methylidyne(5,6-difluoro-3-oxo-1H-indene-2,1(3H)-diylidene)]]bis[propanedinitrile]
**ITIC-4Cl**: 3,9-bis(2-methylene-((3-(1,1-dicyanomethylene)-6,7-dichloro)-indanone))-5,5,11,11-tetrakis(4-hexylphenyl)-dithieno[2,3-d:2′,3′-d′]-s-indaceno[1,2-b:5,6-b′]dithiophene
**Y6**: 2,2′-((2Z,2′Z)-((12,13-bis(2-ethylhexyl)-3,9-diundecyl-12,13-dihydro-[1,2,5]thiadiazolo[3,4-*e*]thieno[2″,3″:4′,5′]thieno[2′,3′:4,5]pyrrolo[3,2-g]thieno[2′,3′:4,5]thieno[3,2-*b*]indole-2,10-diyl)bis(methanylylidene))bis(5,6-difluoro-3-oxo-2,3-dihydro-1H-indene-2,1-diylidene))dimalononitrile
**PDINO**: 2,9-Bis[3-(dimethyloxidoamino)propyl]anthra[2,1,9-def:6,5,10-d′e′f′]diisoquinoline-1,3,8,10(2H,9H)-tetrone
