# Supplementary material for: A Nonionic Alcohol Soluble Polymer Cathode Interlayer Enables Efficient Organic and Perovskite Solar Cells
Source: Chem Mater. 2021 Jul 20;33(22):8602–11. doi: 10.1021/acs.chemmater.1c01430 (PMC8944940; doi:10.1021/acs.chemmater.1c01430)
Supplement: Supplementary file 1 — cm1c01430_si_001.pdf [file cm1c01430_si_001.pdf]

Supporting Information

A Non-ionic Alcohol Soluble Polymer Cathode Interlayer Enables Efficient Organic and Perovskite Solar Cells

Anirudh Sharma,[†] Saumya Singh,[‡] Xin Song,[†] Diego Rosas Villalva,[†] Joel Troughton,[†] Daniel Corzo,[†] Levent Toppare,[ƨ] Gorkem Gunbas[ƨ,*], Bob C. Schroeder[‡,*], and Derya Baran[†,*]

[†]*King Abdullah University of Science and Technology (KAUST), Physical Sciences and Engineering Division (PSE), KAUST Solar Center (KSC), 23955, Thuwal, Saudi Arabia*

[‡]*Department of Chemistry, University College London, London WC1H 0AJ, UK*

[ƨ]*Middle East Technical University (METU), Department of Chemistry, 06800, Ankara, Turkey; ODTU GUNAM, Middle East Technical University, 06800, Ankara, Turkey*

1. SYNTHESIS

1. 1. Monomer synthesis:

2,6-Dibromo-1,4,5,8-naphthalenetetracarboxylic dianhydride ($NDABr_2$)[1] and side chain **1,**[2,3] were synthesized by following the reported protocols.

Oligo ethylene glycol (OEG) side chain (1)

Oligo ethylene glycol (OEG) side chain (1) was synthesized by following the reported protocols. [2,3]

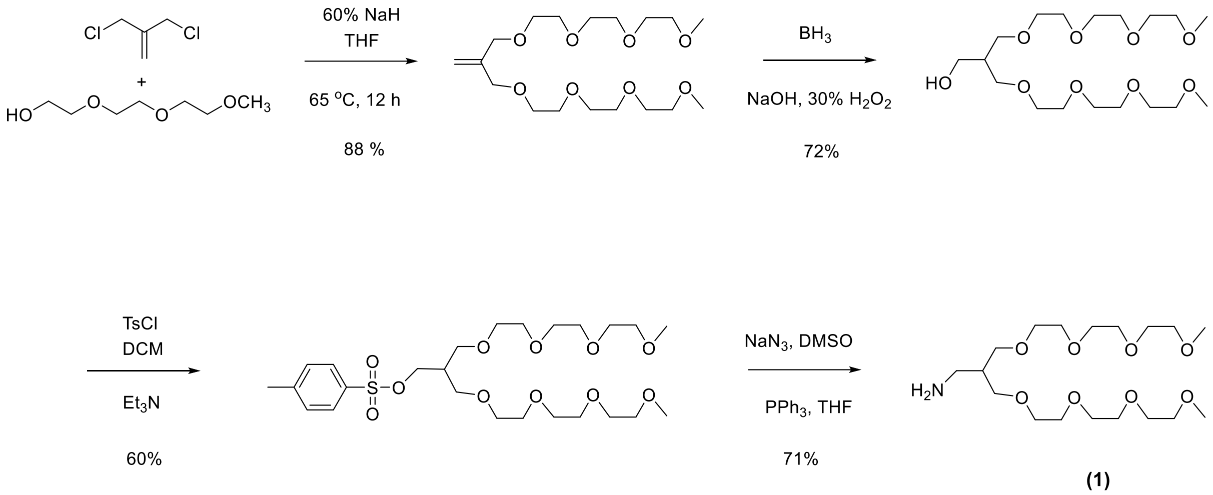

Scheme S1. Synthetic route for branched oligo (ethylene glycol) side chain (**1**).

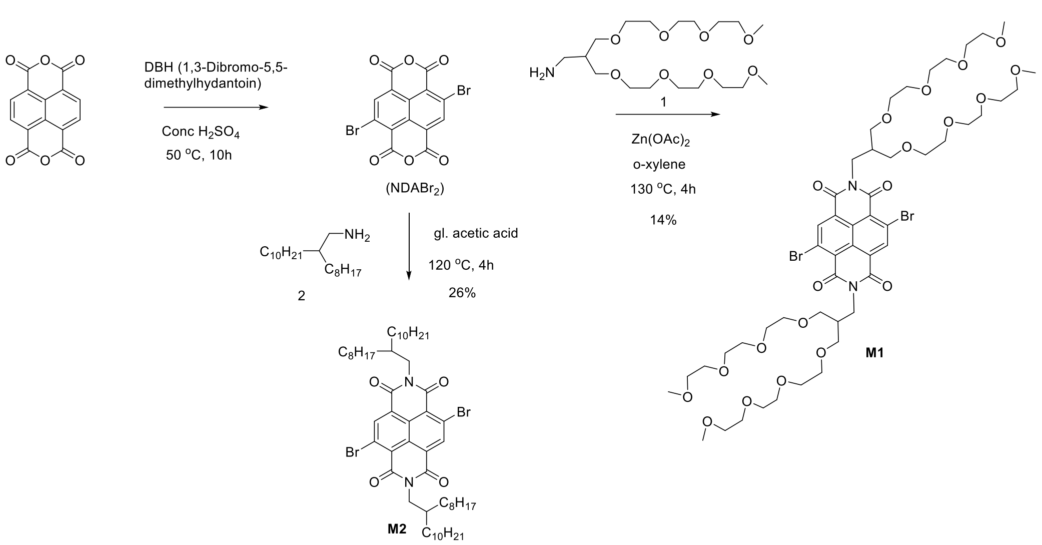

Scheme S2. Synthetic routes for **NDI-Br2** monomers **M1 and M2**.

2,6-dibromoNDI-Teg2 (M1)

2,6-dibromonaphthalene-1,4,5,8-tetracarboxylic dianhydride (NDABr2) (1.00 g, 2.3 mmol), branched OEG amine side chain **1** (1.95 g, 4.9 mmol), and anhydrous zinc acetate (0.862 g, 4.7 mmol) were dissolved in 150 mL of anhydrous o-xylene under inert and dry reaction

conditions. The reaction mixture was heated to 130 °C for 3 hours. The reaction progress was monitored by TLC, and side-chain **1** was further added if required. The reaction was continued at the same temperature for 1 h. After completion, the solvent was removed under reduced pressure. 100 mL of dichloromethane was added to the flask, and the insoluble solid residue was filtered out. Soluble dichloromethane fraction was concentrated under reduced pressure to obtain a sticky product mixture. The crude product was purified by silica gel column chromatography using hexane/ dichloromethane/ acetone/ (5/ 3/ 2 v/v) with the addition of 3-4% of methanol to obtain an orange-yellow solid **M1** with a yield of 14 % (400 mg).

^{1}H NMR (700 MHz, $CDCl_3$) δ ppm: 8.94 (s, 2H), 4.30 (d, *J* = 7.1 Hz, 4H), 3.59 – 3.44 (m, 56H), 3.33 (s, 12 H), 2.54 – 2.48 (m, 2H). ^{13}C NMR (175 MHz, $CDCl_3$) δ ppm: 161.2, 161.1, 139.1, 128.3, 127.9, 125.5, 124.3, 72.1, 71.1, 70.7 (multiple peaks), 70.6, 70.5, 59.2, 42.0, 38.5.

ESI MS: 1185.3419 $[M+H]^+$ (calc. 1185.3410)

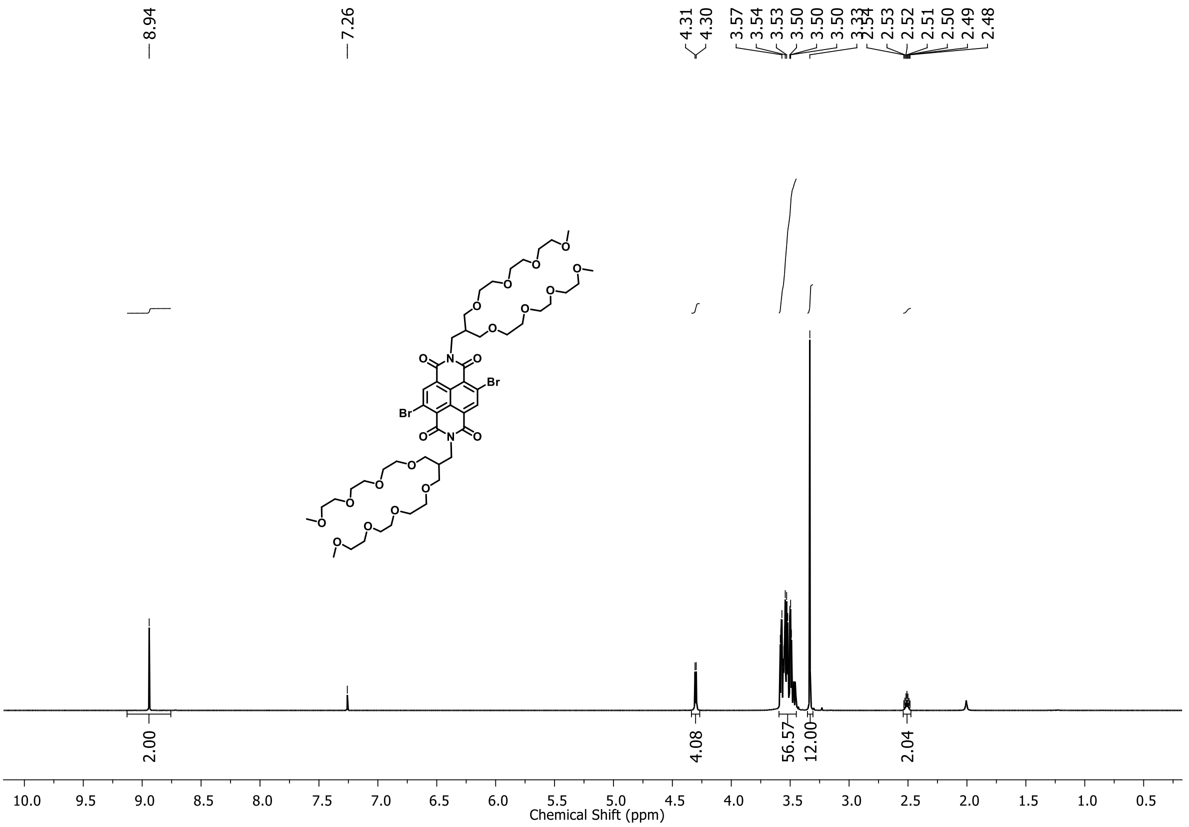

Figure S1: ^{1}H NMR spectrum of **M1** in $CDCl_3$.

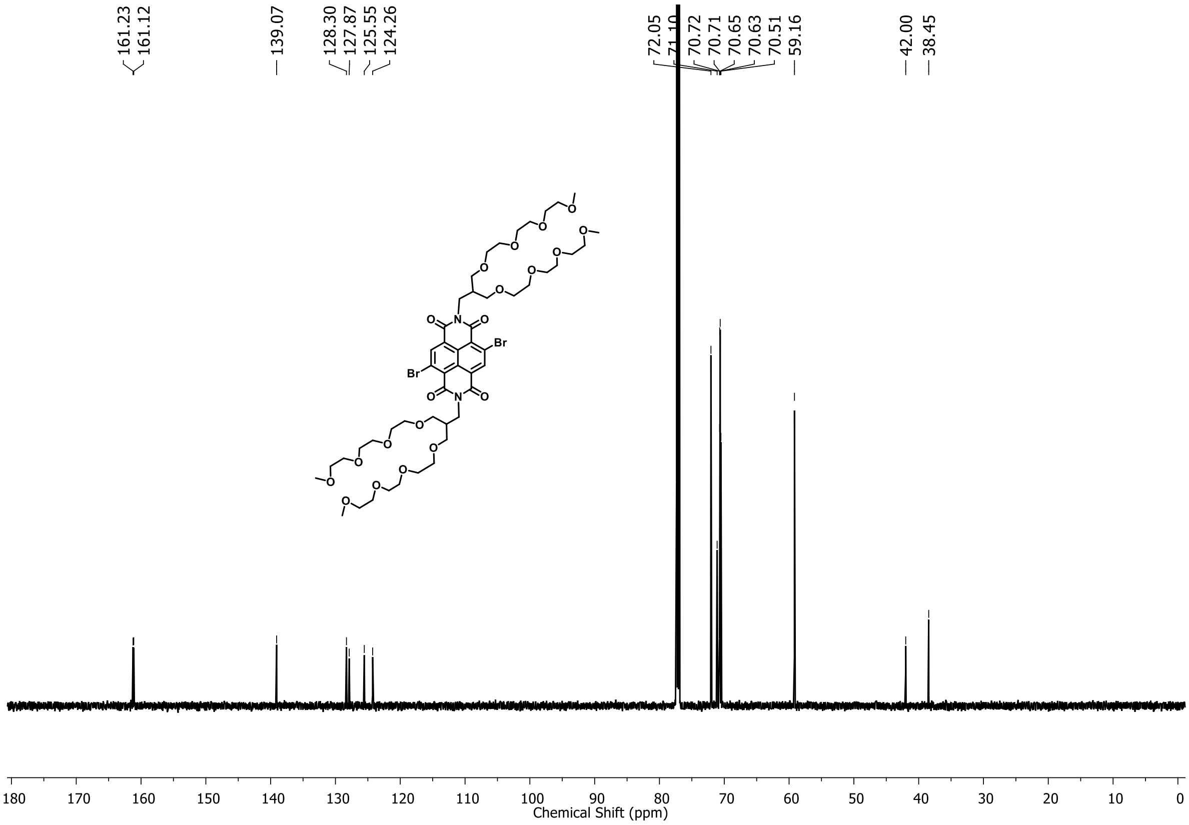

Figure S2: ^{13}C NMR spectrum of **M1** in $CDCl_3$.

NDI-OD-Br$_2$ (M2)

2,6-dibromonaphthalene-1,4,5,8-tetracarboxylic dianhydride (NDABr$_2$) (5.00 g, 11.7 mmol) was suspended in 100 mL of glacial acetic acid and was stirred for 10-15 min to get a homogeneous dispersion. 2-octyldodecyl amine (8.73 g, 29.3 mmol) was added to the reaction flask, and the reaction mixture was heated at 120 °C for 3 h. After completion, the reaction mixture was concentrated under reduced pressure and then precipitated into methanol to get a reddish-brown powder. The crude product was purified by column chromatography using a pet ether/ethyl acetate solvent system and then re-crystallized from a 1:1 mixture of hexane and acetone to get a yellow powder **M2** with a yield of 26 % (3.0 g).

^{1}H NMR (400 MHz, $CDCl_3$) δ ppm: 8.97 (s, 2H), 4.13 (d, *J* = 7.4 Hz, 4H), 1.97 (m, 2H), 1.39 – 1.22 (m, 64 H), 0.88 – 0.83 (m, 12H). ^{13}C NMR (100 MHz, $CDCl_3$) δ ppm: 161.2, 161.1, 139.2, 128.4, 127.8, 125.3, 124.1, 45.5, 36.5, 32.0, 31.9, 31.6, 30.1, 29.7 (multiple peaks), 29.6, 29.4, 29.4, 26.4, 22.8, 22.7, 14.2. ESI MS: 984.48 (calc. 984.37)

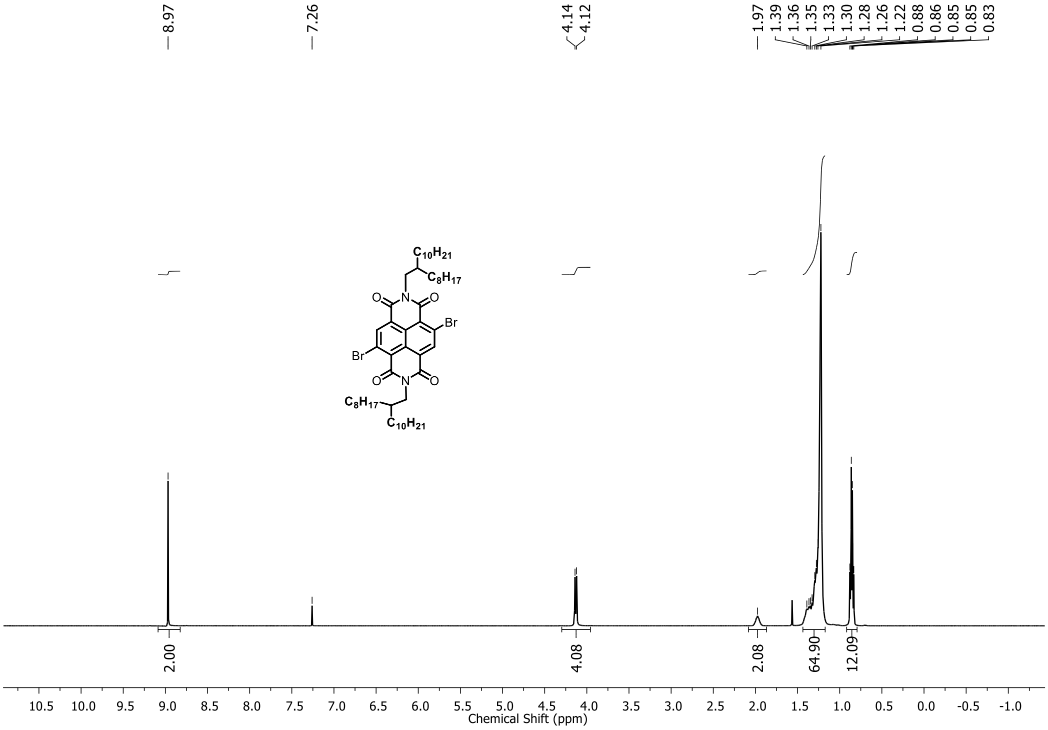

Figure S3: ^{1}H NMR spectrum of **M2** in $CDCl_3$.

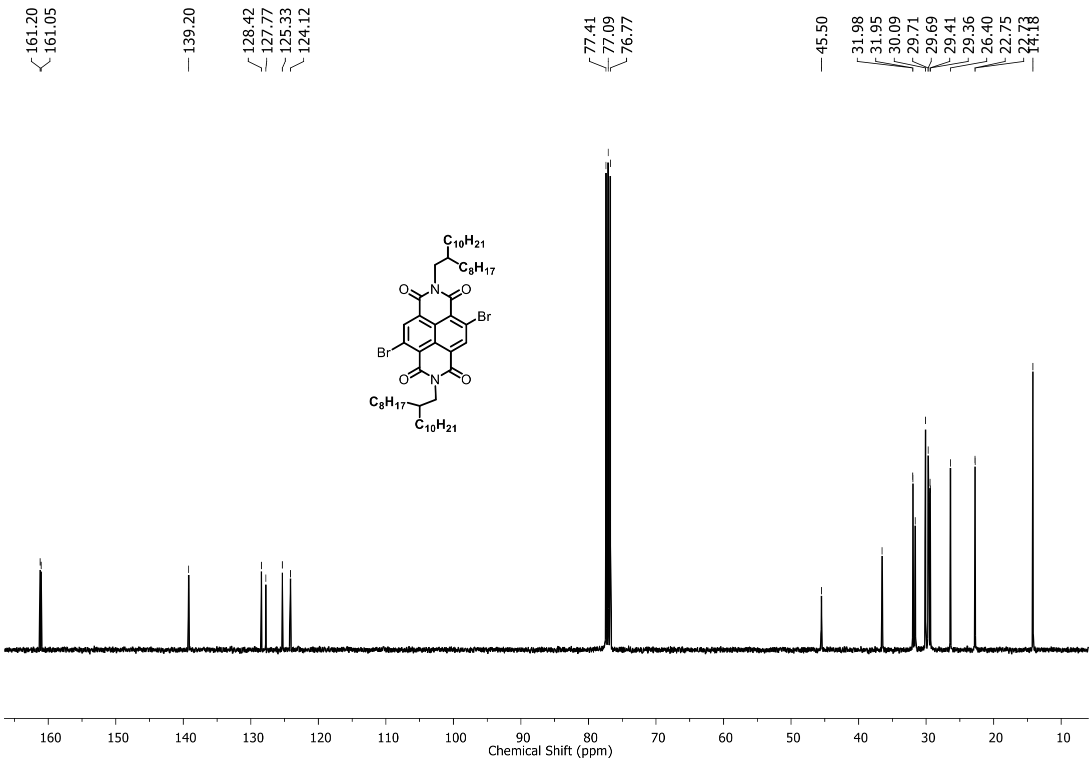

Figure S4: ^{13}C NMR spectrum of **M2** in $CDCl_3$.

1.2. Polymer Synthesis:

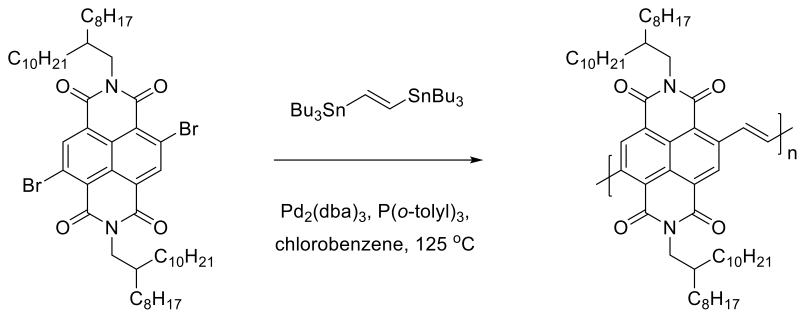

Scheme S3. Synthesis of alkyl side chain substituted polymer P2.

Polymer P2: In a 20 mL microwave vial, monomers N,N'-bis(2-octyldodecyl)-2,6-dibromonaphthalene-1,4,5,8-bis(dicarboximide) (**M2**, 400 mg, 0.406 mmol) and trans-1,2-Bis(tributylstannyl)ethene (246 mg, 0.406 mmol) were dissolved in 15 mL of anhydrous, degassed chlorobenzene. $Pd_2(dba)_3$ (2 mol%) and P(*o*-tol)$_3$ (8 mol%) were added to the solution, and the reaction mixture was purged with N_2 for 10 min. The vial was sealed and heated to 125 °C for 48 hours, followed by the end-capping procedure. After end-capping, the reaction mixture was cooled to room temperature. The crude reaction mixture was then precipitated in cold methanol. The solid obtained was directly filtered into a glass-fiber thimble and washed with cold hexane: acetone (1:1) solvent mixture to remove monomers and oligomers. Soxhlet extraction was carried out with methanol for 12 h followed by acetone and then a solvent mixture of hexane: methanol (1:4) to remove unreacted monomers and oligomers. Final extraction was done with chloroform to collect polymer. The chloroform fraction was then passed through a short celite plug to remove the catalyst. The chloroform fraction was further concentrated under reduced pressure and precipitated from cold methanol. **P2** (220 mg, 63.5%) ^{1}H NMR (400 MHz, $CDCl_3$) δ: 9.23 (broad s, 2H), 8.97 (broad s, 2H), 4.20 (broad s, 4H), 2.08 (broad s, 2H), 1.40- 1.21 (m, 64H), 0.88- 0.82 (m, 12H)

GPC (dichlorobenzene, 80 °C): M_n = 11,680 g·mol^{-1}, M_w = 17,449 g·mol^{-1}, and Đ = 1.49

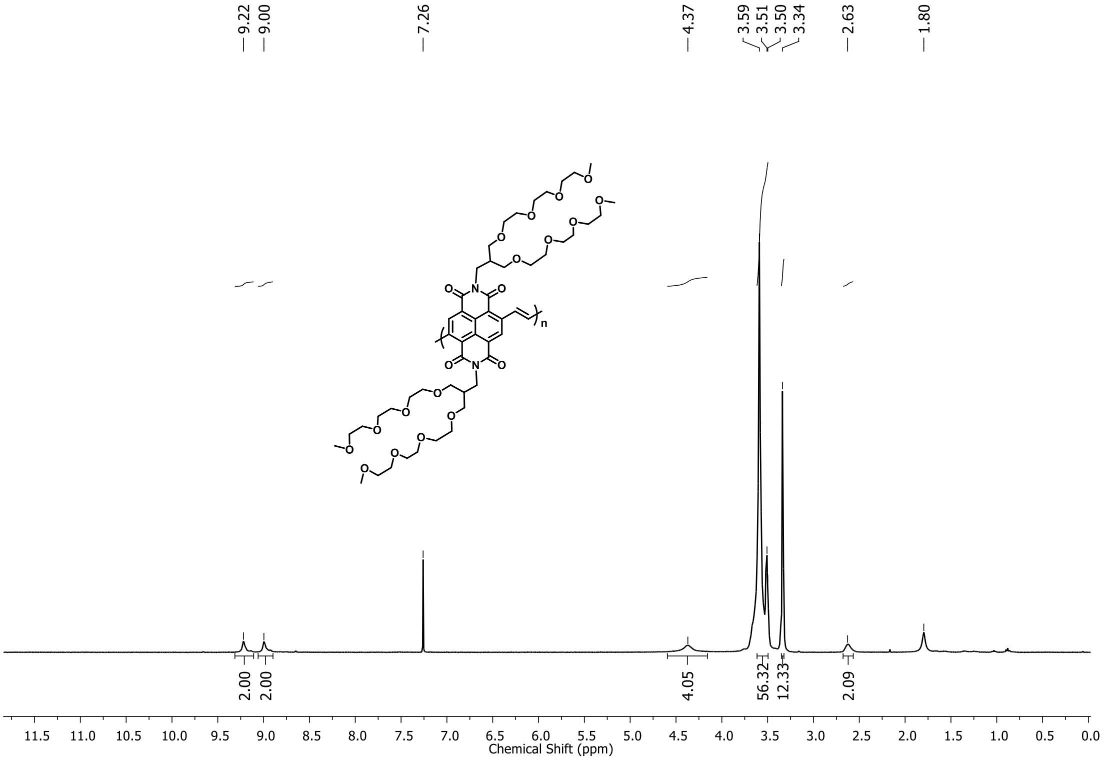

Figure S5: ^1H NMR spectrum of OEG side-chain polymer P2G in $CDCl_3$.

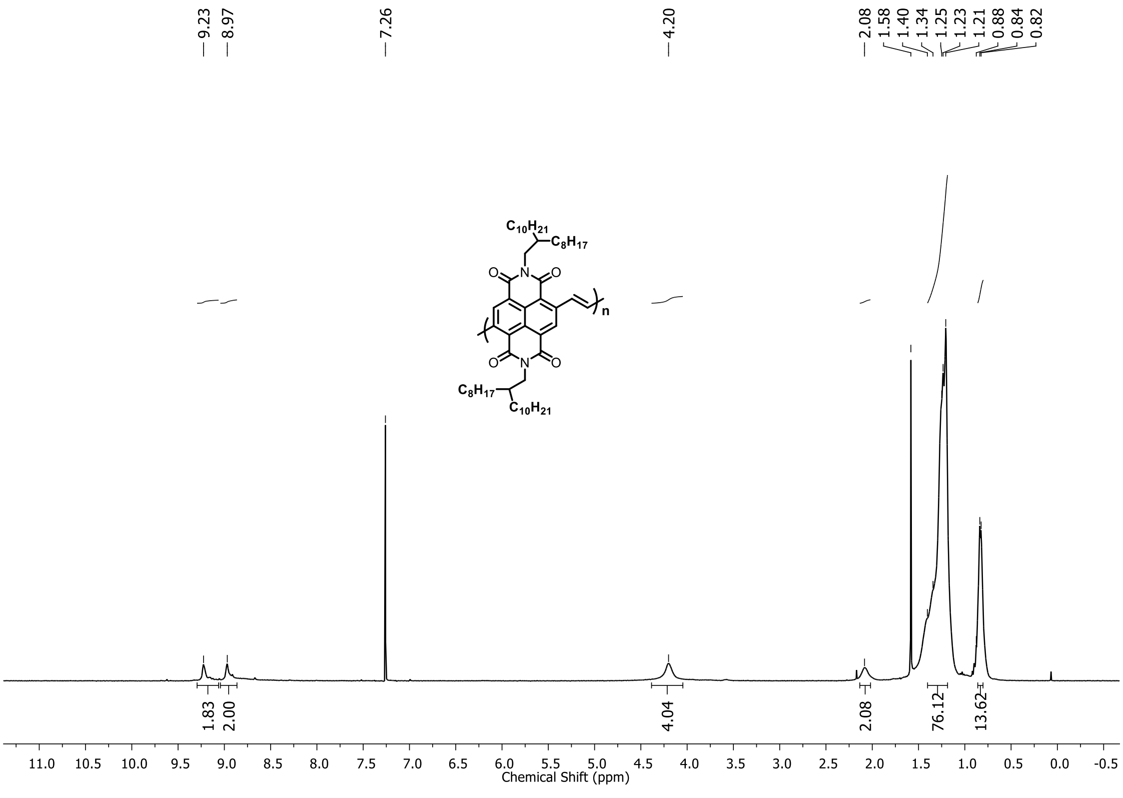

Figure S6: ^1H NMR spectrum of alkyl side chain polymer P2 in $CDCl_3$.

2. MATERIALS AND DEVICE CHARACTERISATION

Cyclic voltammetry

For the experiment, a glassy carbon electrode was used as a working electrode, Pt wire as a counter electrode, Ag/ Ag^{+} electrode as the standard electrode, and Fc/ Fc^{+} as an internal standard. **P2G** (10 mg mL^{-1}) and tetrabutylammonium hexafluorophosphate (0.1M) were dissolved in degassed anhydrous dichloromethane and purged with nitrogen for 10 minutes. The first reduction onset potential was recorded to calculate the lowest unoccupied molecular orbital (LUMO) energy level (Figure S4). The onset of reduction of P2G was found to be -0.62 V. LUMO energy levels were calculated following the E_{LUMO} = -(4.8+ E_{red}) eV, and calibrated with respect to the internal standard Fc/Fc+ couple.

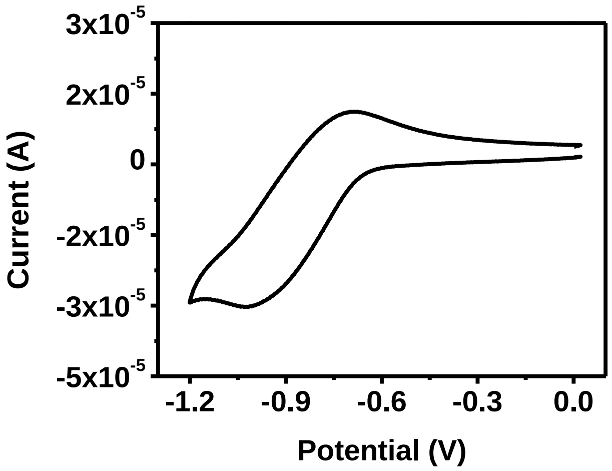

Figure S7: Cyclic voltammogram of polymer P2G at a scan rate of 100 mV/s in reduction only scans.

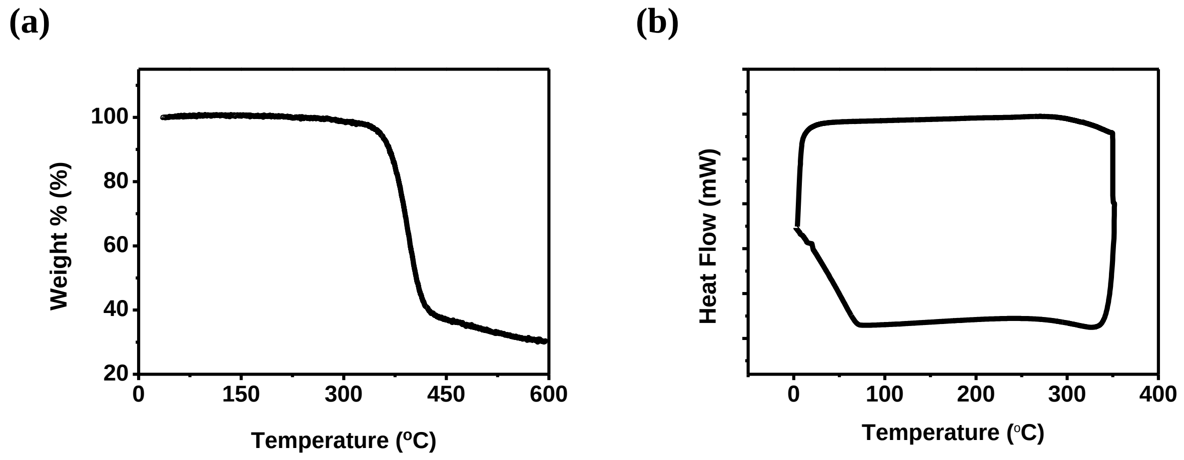

Figure S8: (a) Thermogravimetric analysis (TGA) curves for polymers P2G and (b) Differential scanning calorimetry (DSC) measurement of the polymer P2G at a heating rate of 10 °C min^{-1} under a nitrogen atmosphere (the second cycle is shown).

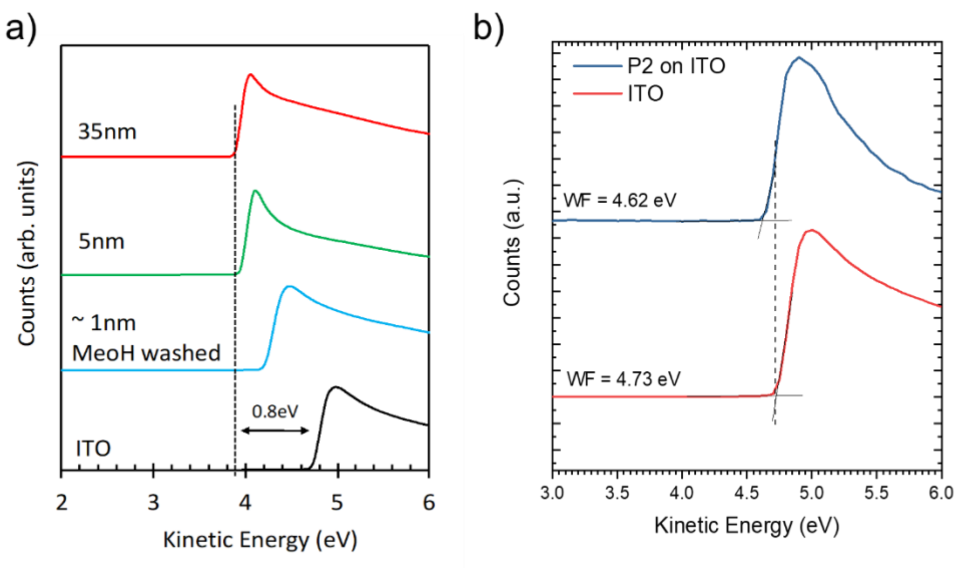

Figure S9: Secondary electron cut-off of the UPS spectra depicting the change in the ITO work function a) with the P2G thickness b) with P2.

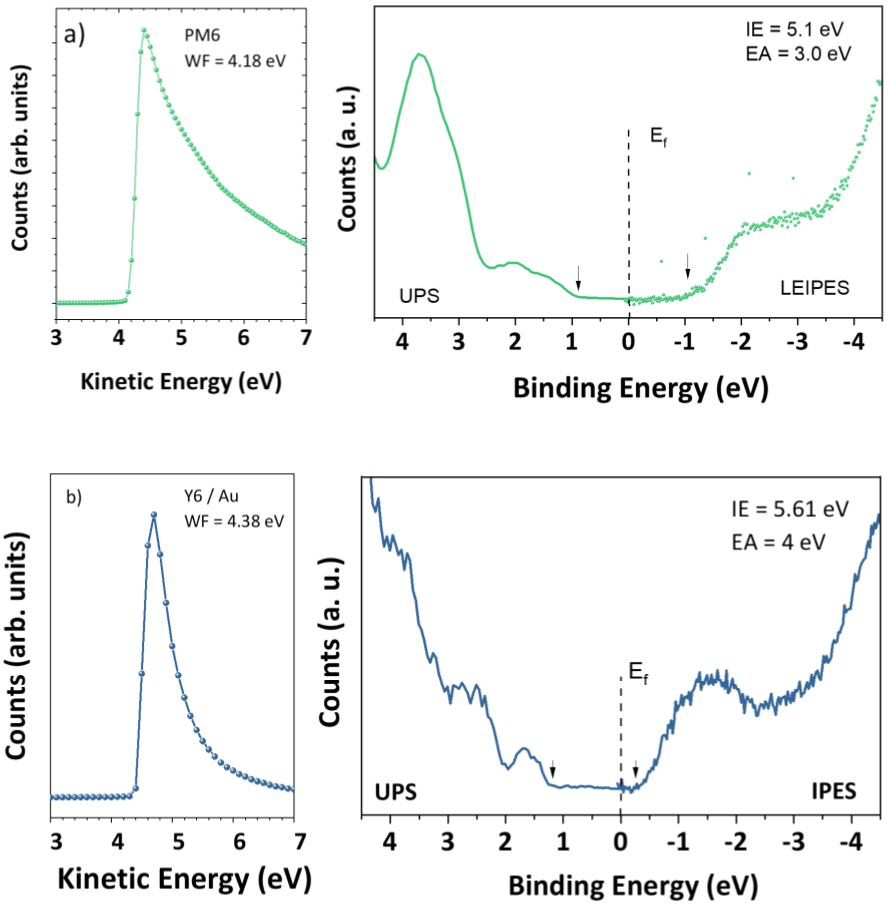

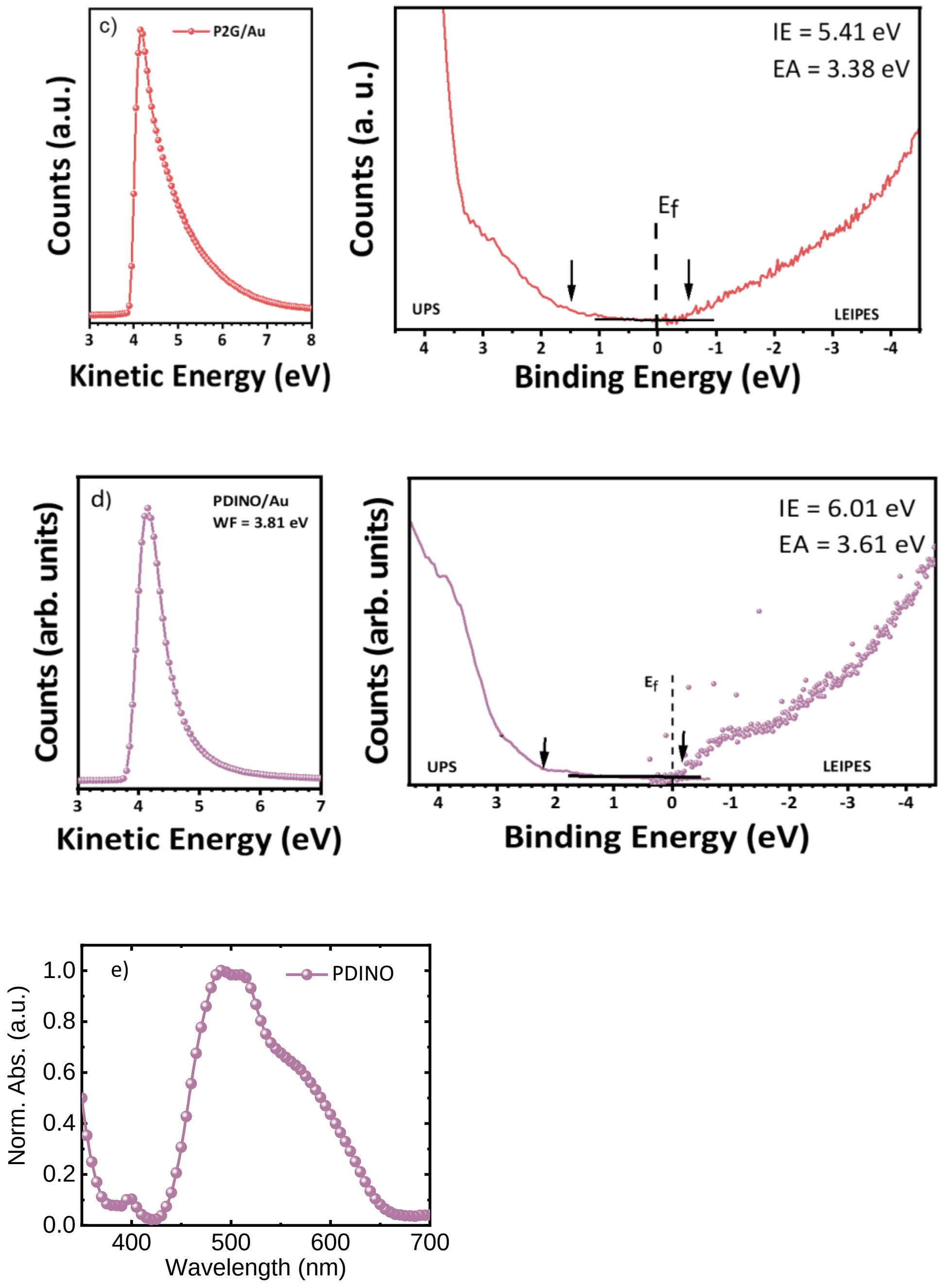

Figure S10: UPS and LEIPES spectra of a) PM6 b) Y6 c) P2G d) PDINO, showing the work function, HOMO, and LUMO onsets. e) UV-vis absorption spectra of PDINO thin film.

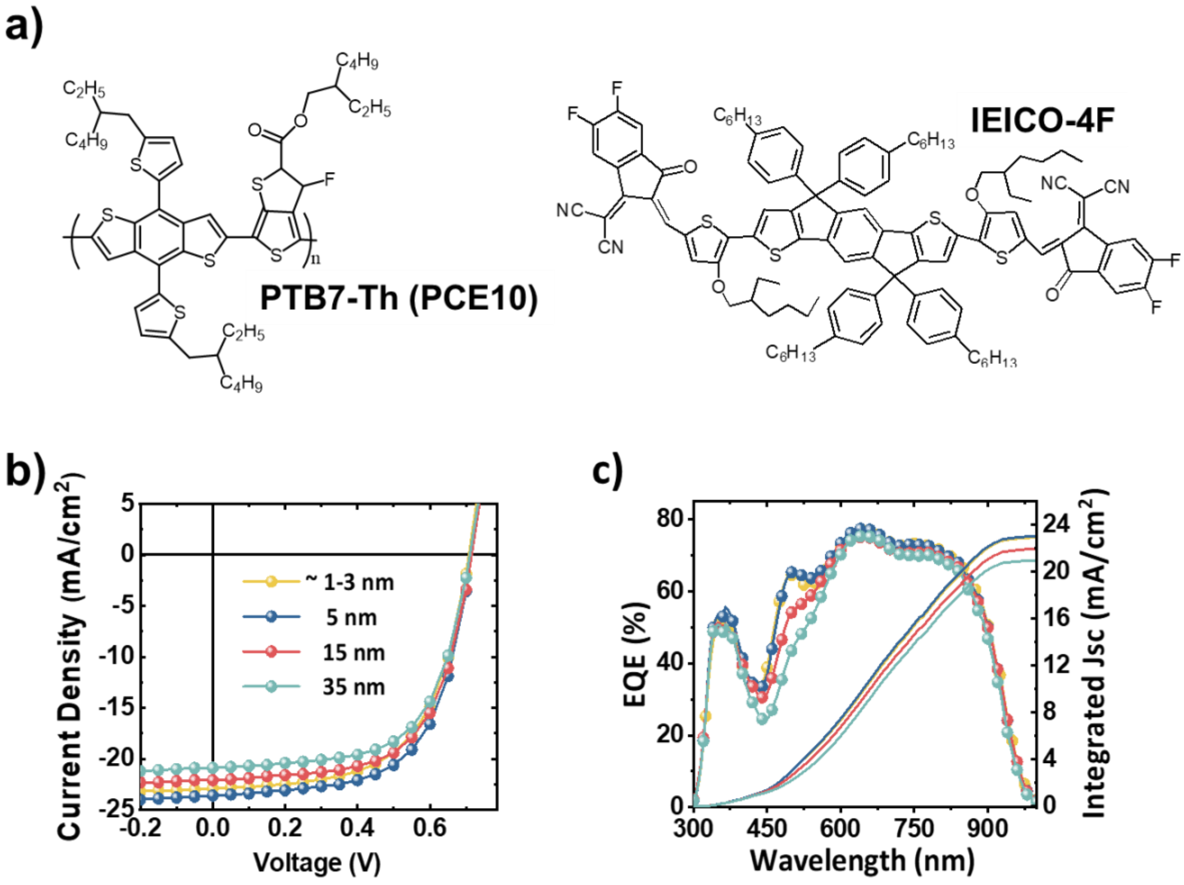

Figure S11: a) Chemical structure of PTB7-Th and IEICO-4F b) J-V and c) EQE curves of PTB7-Th: IEICO-4F devices with the P2G *CIL* of varying thickness.

Devices with Inkjet-Printed P2G *CIL*

The device stack - ITO/PEDOT: PSS/PTB7-Th: IEICO4F was prepared identically to that reported in the main text for spin-coated devices. The P2G *CIL* was then inkjet-printed (0.1mg/mL solution in a mixture of ethanol, methanol, and pentanol (1:1:1)) with a drop spacing of 20µm, and dried at room temperature. For best performing devices, 3 layers for P2G were printed over one another to achieve the optimum thickness. 100 nm of Ag was then evaporated as the metal electrode, defining the active area to be 0.1 cm^2.

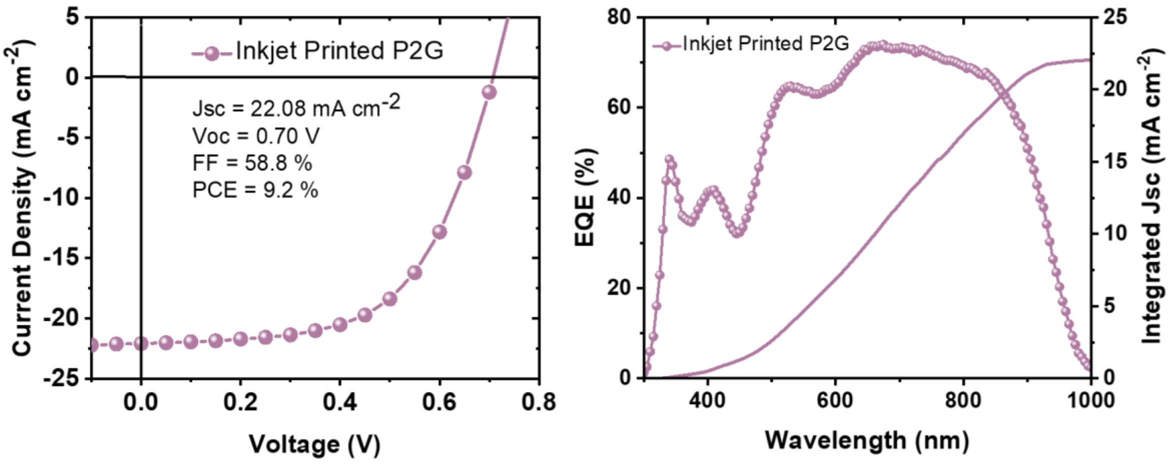

Figure S12: J-V curve of PTB7-Th: IEICO4F device with inkjet-printed P2G *CIL*

PhotoStability of Devices with P2G and PNDIO as the CIL

The photostability study was conducted in a home-built setup under inert conditions maintained with a constant flow of nitrogen. The samples were irradiated under LED lamp at 1sun condition and maintained at a temperature of under 38° C using a cooling system.The J-V curves were tracked at regular intervals of time to follow the performance parameters of the device.

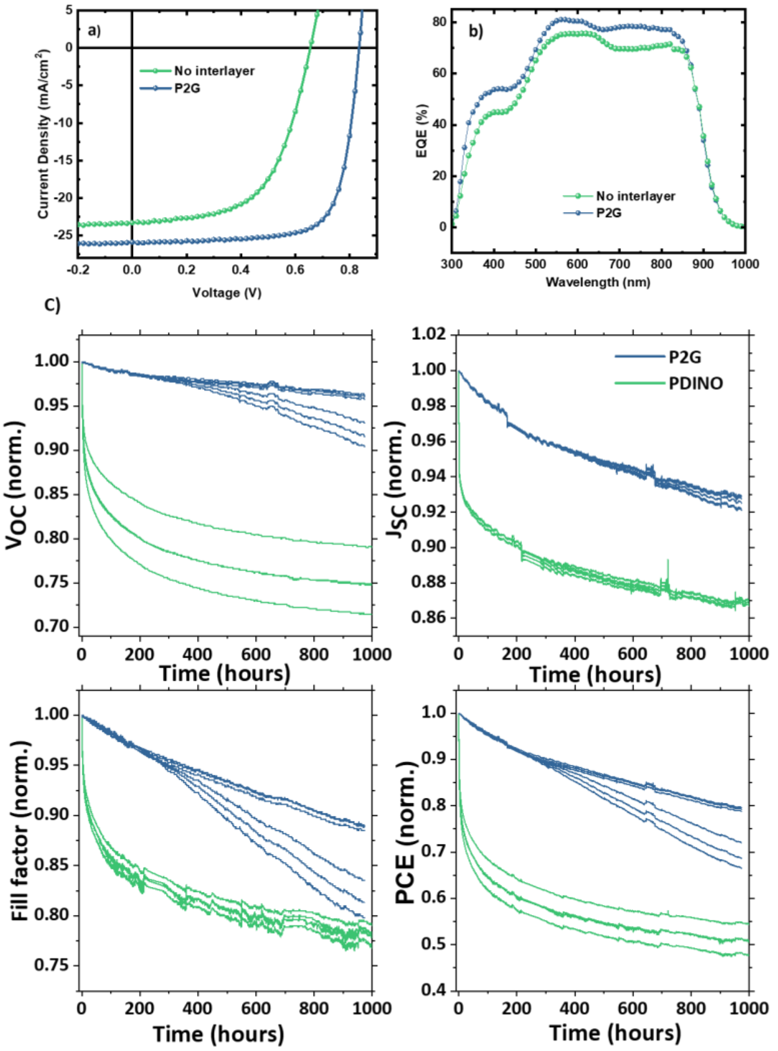

Figure S13: a) J-V and b) EQE plots of PM6:Y6 devices with and without P2G. c) photo-stability of PM6:Y6 devices using P2G and PDINO as the CIL, demonstrating the changes in the device parameters: Jsc, FF, Voc and PCE under one sun conditions.

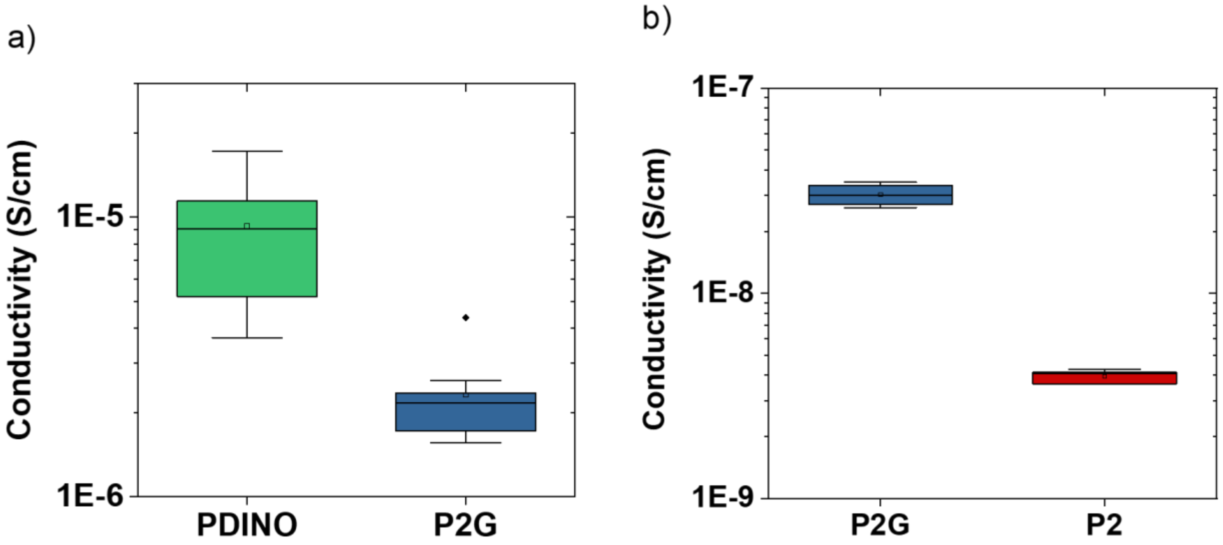

Figure S14: The electrical conductivity of a) PDINO and P2G processed from methanol b) P2G and P2 processed from chloroform.

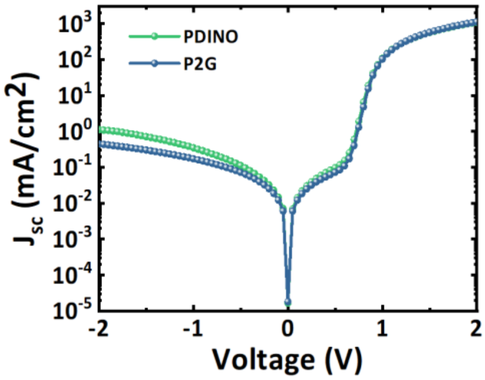

Figure S15: Current-voltage measurements of PM6:Y6 devices with P2G and PDINO *CILs* under dark condition

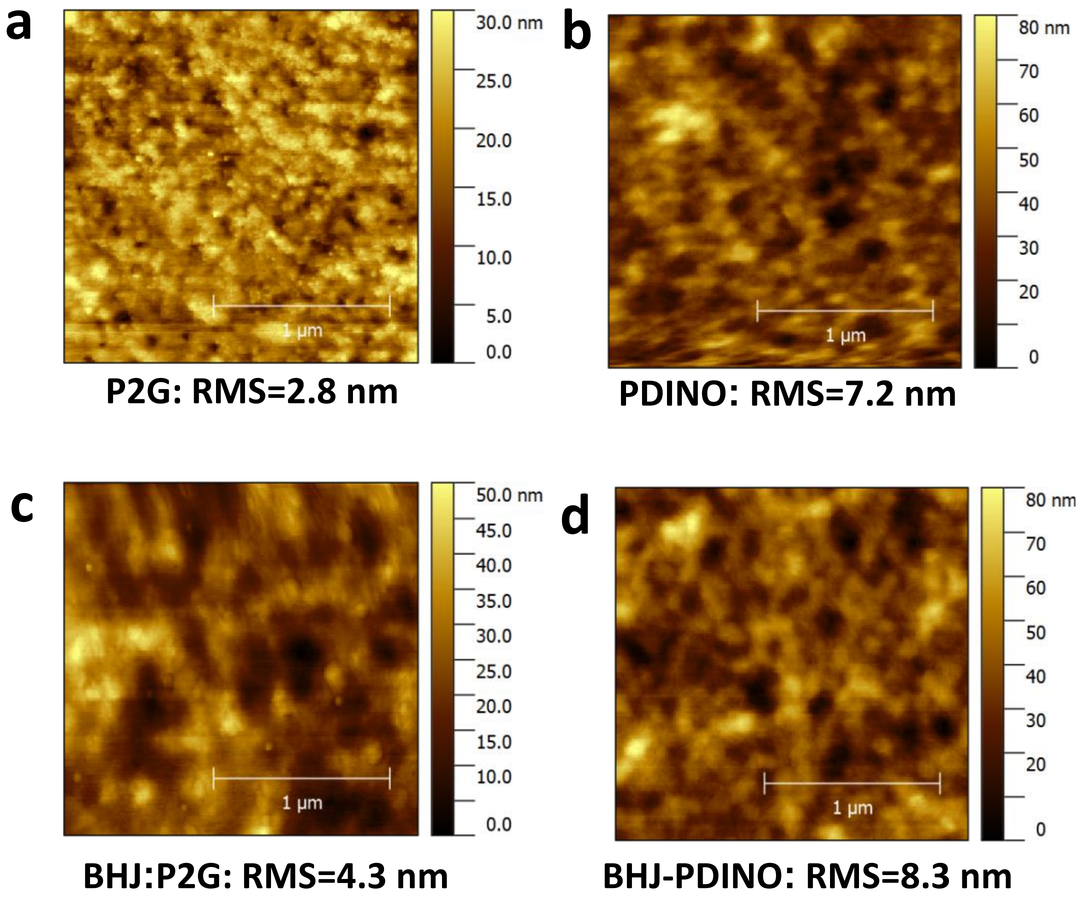

Figure S16. Tapping-mode *AFM* topography of (a) neat P2G (b) PDINO (c) PM6:Y6 active layer-P2G and (d) PM6:Y6 active layer-PDINO.

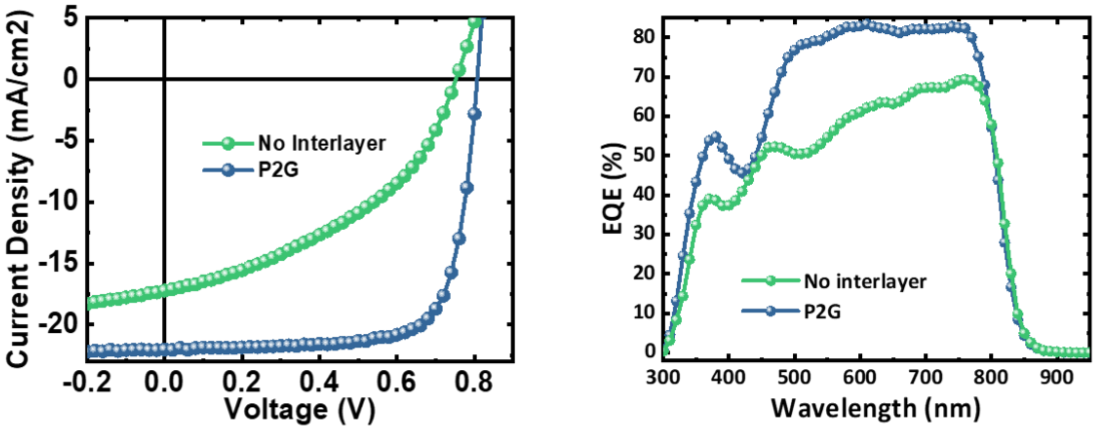

Figure S17: J-V and EQE measurements of PM6:ITIC-4Cl devices with and without P2G *CIL*

Table S1: Photovoltaic parameters of PM6-ITIC4Cl devices with and without PNDI-GV under the illumination of AM 1.5 G, 100 mW cm^{-2}. Average values calculated over ten devices.

PM6:IT-4Cl	Jsc [mA/cm-2]	Voc [V]	FF [%]	PCE [%]	Average PCE [%]
P2G	22.1	0.81	74.7	13.3	12.8
No interlayer	17.2	0.75	42.6	5.50	5.21

Table S2. Photovoltaic parameters of perovskite (MAPI) solar cells with and without P2G interlayer under AM1.5G illumination (100 mW cm^{-2}). Average *PCE* values are from six devices.

Perovskite	J_{sc} [mAcm^{-2}]	Integrated J_{sc} [mA cm^{-2}]	V_{oc} [V]	FF [%]	PCE [%]	Ave. PCE [%]
No interlayer	22.0	21.6	1.04	65	15.0	14.6
P2G	22.1	22.0	1.04	77	17.6	17.1

REFERENCES

1. Sarkar, A.; Dhiman, S.; Chalishazar, A.; George, S. J. Visualization of Stereoselective Supramolecular Polymers by Chirality-Controlled Energy Transfer. *Angew. Chem. Int. Ed.* **2017,** *56*, 13767-13771.
2. Zhu, S.; Zhang, J.; Vegesna, G.; Luo, F.-T.; Green, S. A.; Liu, H. Highly Water-Soluble Neutral BODIPY Dyes with Controllable Fluorescence Quantum Yields. *Org. Lett.* **2011,** *13*, 438-441.
3. Wu, J.; You, L.; Lan, L.; Lee, H. J.; Chaudhry, S. T.; Li, R.; Cheng, J.-X.; Mei, J. Semiconducting Polymer Nanoparticles for Centimeters-Deep Photoacoustic Imaging in the Second Near-Infrared Window. *Adv. Mater.* **2017,** *29*, 1703403.
